# Genome-wide identification of RNA editing in seven porcine tissues by matched DNA and RNA high-throughput sequencing

**DOI:** 10.1186/s40104-019-0326-9

**Published:** 2019-03-13

**Authors:** Yuebo Zhang, Longchao Zhang, Jingwei Yue, Xia Wei, Ligang Wang, Xin Liu, Hongmei Gao, Xinhua Hou, Fuping Zhao, Hua Yan, Lixian Wang

**Affiliations:** Key Laboratory of Animal (Poultry) Genetics Breeding and Reproduction, Ministry of Agriculture; Institute of Animal Science, Chinese Academy of Agricultural Sciences, Beijing, 100193 China

**Keywords:** ADAR, A-to-G, High-throughput sequencing, RNA editing, Swine

## Abstract

**Background:**

RNA editing is a co/posttranscriptional modification mechanism that increases the diversity of transcripts, with potential functional consequences. The advent of next-generation sequencing technologies has enabled the identification of RNA edits at unprecedented throughput and resolution. However, our knowledge of RNA editing in swine is still limited.

**Results:**

Here, we utilized RES-Scanner to identify RNA editing sites in the brain, subcutaneous fat, heart, liver, muscle, lung and ovary in three 180-day-old Large White gilts based on matched strand-specific RNA sequencing and whole-genome resequencing datasets. In total, we identified 74863 editing sites, and 92.1% of these sites caused adenosine-to-guanosine (A-to-G) conversion. Most A-to-G sites were located in noncoding regions and generally had low editing levels. In total, 151 A-to-G sites were detected in coding regions (CDS), including 94 sites that could lead to nonsynonymous amino acid changes. We provide further evidence supporting a previous observation that pig transcriptomes are highly editable at PRE-1 elements. The number of A-to-G editing sites ranged from 4155 (muscle) to 25001 (brain) across the seven tissues. The expression levels of the ADAR enzymes could explain some but not all of this variation across tissues. The functional analysis of the genes with tissue-specific editing sites in each tissue revealed that RNA editing might play important roles in tissue function. Specifically, more pathways showed significant enrichment in the fat and liver than in other tissues, while no pathway was enriched in the muscle.

**Conclusions:**

This study identified a total of 74863 nonredundant RNA editing sites in seven tissues and revealed the potential importance of RNA editing in tissue function. Our findings largely extend the porcine editome and enhance our understanding of RNA editing in swine.

**Electronic supplementary material:**

The online version of this article (10.1186/s40104-019-0326-9) contains supplementary material, which is available to authorized users.

## Background

In the 1980s, Benne et al. discovered an RNA editing event in which four nonencoded nucleotides were inserted into the mRNA of the mitochondrial cytochrome oxidase subunit II (*coxII*) gene in trypanosomatids [1]. Subsequently, RNA editing was defined as a co/posttranscriptional modification mechanism that alters sequence information at the RNA level by introducing differences between a final RNA sequence and its template DNA through the insertion, deletion or substitution of nucleotides [2]. This modification can occur in coding regions (CDS) and noncoding regions, thereby recoding amino acids, affecting alternative splicing, influencing RNA stability, and modulating the nuclear retention of RNAs [3, 4]. In mammals, adenosine-to-inosine (A-to-I) editing catalyzed by adenosine deaminases acting on RNA (ADAR) is the most common type of RNA editing [5, 6]. As inosine is generally read as guanosine (G) by the cellular machinery, A-to-I editing is also named A-to-G editing. The ADAR enzyme family, which primarily includes three members (ADAR1, ADAR2 and ADAR3), targets only double-stranded RNA to catalyze A-to-G RNA editing [7]. Both ADAR1 and ADAR2 are essential for normal development, and a homozygous null mutation in either of these two genes causes early lethality in mice [8, 9].

The identification of RNA editing sites heavily depends on sequencing technologies. Therefore, RNA edits were originally regarded as rare variants due to the limitations of sequencing technologies [10]. The advent of next-generation sequencing (NGS) technologies has enabled the identification of transcriptome-wide RNA editing events across individuals and tissues at unprecedented throughput and resolution. Subsequently, the discovery rate of RNA editing sites has dramatically increased. With the development of bioinformatics tools designed for RNA editing detection and the significant decline in sequencing costs, RNA editing studies are ushering in an unprecedented opportunity. Currently, RNA editing studies are widely implemented in humans and mice by comparing matched RNA and DNA sequencing data or using only RNA sequencing (RNA-seq) data [11–19]. Moreover, separate laboratories have reported the potentially biological significance of RNA editing in disease pathobiology and tumorigenicity [20, 21], introducing a new regulatory layer to enhance our understanding of complex diseases. Pigs are considered an ideal animal model of human diseases since they share similar anatomic and physiologic characteristics with humans. Therefore, RNA editing studies involving pigs can promote an understanding of the molecular basis of human diseases. However, to the best of our knowledge, only one research study, which identified 5294 nonredundant A-to-G sites across three tissues from one pig, has provided information about RNA editing at a transcriptome-wide level in pigs [22]. Our knowledge of RNA editing in pigs is very limited compared to that in humans and other model species.

Given the significance of pigs in biomedical research and animal husbandry, we systematically detected and characterized the RNA editome in pigs based on strand-specific RNA sequencing data and whole-genome sequencing data of the brain, fat, heart, liver, lung, muscle and ovary from three 180-day-old Large White gilts. We revealed a total of 74863 RNA editing sites and implemented a detailed characterization of the sequence and distribution features of these sites. We found frequent occurrence in noncoding regions, especially PRE-1, providing further evidence supporting a previous observation that pig transcriptomes are highly editable in PRE-1 elements. Furthermore, the functional analysis of the genes with tissue-specific editing sites in each tissue revealed the potential functional importance of RNA editing in porcine tissue regulation. Our study largely extends the list of RNA editing sites in swine and provides deeper insight into the characteristics of the pig editome.

## Methods

### Sample collection and nucleic acid isolation

The frontal lobe of the brain, tip of the heart, left lateral lobe of the liver, caudal lobe of the left lung, *Longissimus dorsi* (the 10-11^th^ rib, muscle), follicles and surrounding tissue of the ovary, and shoulder subcutaneous fat were collected from three 180-day-old Large White gilts (Table 1 and Additional file 1: Figure S1), snap frozen in liquid nitrogen and stored at − 80 °C until use. The genomic DNA was extracted from the muscle samples by using the standard phenol-chloroform protocol. The total RNA was isolated using TRIzol reagent (Invitrogen, Carlsbad, CA, USA) according to the manufacturer’s instructions. Only DNA samples with OD 260/280 ratios of 1.8~2.0 and total contents greater than 1.5 μg were used in the subsequent steps. RNA samples with RNA integrity number (RIN) scores higher than seven were used in this study.

**Table 1:** Statistics of the high-throughput sequencing

Sample name	Tissue type	Individual	RNA-seq		DNA-seq		
			Total reads	Total mapped rate	Total reads	Mapping rate	Coverage^a^
Brain1	Brain	1	65,772,910	86.7%			
Brain2	Brain	2	77,952,110	87.9%			
Brain3	Brain	3	102,517,764	87.9%			
Fat1	Fat	1	71,195,080	83.9%			
Fat2	Fat	2	74,251,406	85.2%			
Fat3	Fat	3	69,499,032	85.0%			
Heart1	Heart	1	69,228,312	88.1%			
Heart2	Heart	2	74,663,108	87.0%			
Heart3	Heart	3	71,989,840	87.9%			
Liver1	Liver	1	69,832,472	87.8%			
Liver2	Liver	2	66,986,370	86.9%			
Liver3	Liver	3	75,826,328	88.4%			
Lung1	Lung	1	68,902,820	85.8%			
Lung2	Lung	2	71,797,208	84.3%			
Lung3	Lung	3	76,766,772	84.7%			
Muscle1	Muscle	1	72,955,650	79.2%	360,404,542	87.2%	85.9%
Muscle2	Muscle	2	73,898,278	81.7%	476,837,820	88.4%	81.2%
Muscle3	Muscle	3	79,972,012	80.0%	500,781,658	88.8%	82.1%
Ovary1	Ovary	1	68,906,170	85.6%			
Ovary2	Ovary	2	86,181,796	86.9%			
Ovary3	Ovary	3	82,193,394	86.3%			
Average			74,823,278	85.6%	446,008,007	88.1%	83.1%

^a^The coverage was estimated based autosomal and X chromosomes

### Strand-specific transcriptome sequencing

Three micrograms of total RNA per sample were used for the subsequent creation of strand-specific RNA-seq libraries. In total, 21 strand-specific sequencing libraries were generated using mRNA purified from total RNA using oligo(dT) beads by NEBNext® Ultra™ Directional RNA Library Prep Kit for Illumina® (NEB, USA) following the manufacturer’s protocol. After qualification by an Agilent 2100 Bioanalyzer and real-time PCR, each library preparation was sequenced on an Illumina HiSeq platform by the Novogene Bioinformatics Technology Cooperation (Beijing, China), and 150 bp paired-end reads were generated.

### Whole-genome sequencing

Muscle DNA was extracted from the three pigs mentioned above and used to construct three DNA libraries (Additional file 1: Figure S1). These libraries were generated using a TruSeq Nano DNA HT Sample Preparation Kit (Illumina, USA) according to the manufacturer’s recommendations. The quantification and quality of the sequencing libraries were assessed by real-time PCR and an Agilent Bioanalyzer 2100 system. Then, each library constructed above was sequenced on an Illumina HiSeq platform provided by the Novogene Bioinformatics Technology Cooperation (Beijing, China), and 150 bp paired-end reads were generated for further analysis.

### Read processing

To ensure the reliability of the reads and reduce the inherent noise of high-throughput sequencing in further analysis, the raw data were first filtered by eliminating the reads containing an adapter or poly-N and low-quality reads through a series of in-house Perl scripts used for quality control (QC). All downstream analyses were based on high-quality filtered data.

### RNA editing detection

The detection of RNA editing sites was conducted using RES-Scanner [23]. Parameters were adopted according to the author’s recommendation. The reference genome *Sus scrofa* 10.2.87 was downloaded from Ensembl (ftp://ftp.ensembl.org/pub/release-87/fasta/sus_scrofa/dna/). The genomic feature position files and Sus_scrofa.dbSNP145.gtf were prepared using custom scripts on the basis of Sus_scrofa.Sscrofa10.2.87.gtf (ftp://ftp.ensembl.org/pub/release-87/gtf/sus_scrofa/) and Sus_scrofa.vcf (ftp://ftp.ensembl.org/pub/release-87/variation/vcf/sus_scrofa/) following the RES-Scanner user manual.

A candidate RNA editing site must meet the following conditions: 1) the genomic site is homozygous with a Bayesian probability exceeding 0.95 and is supported by at least 10 reads; 2) at least three RNA reads differ from the genomic genotype; 3) the site has an editing level ≥ 0.05 and must be supported by at least one RNA read in the middle of its length; 4) the binomial test false discovery rate (FDR) of this site must be < 0.05; and 5) the site is not located within homopolymeric regions of five or more residues and within six intronic bases of a splice site. All thresholds used for the identification of the RNA editing sites were the default parameters of RES-Scanner.

### Validation of RNA editing sites through sanger sequencing

In total, 64 editing sites randomly selected from all sites identified in Brain3 were used to assess the reliability of RES-Scanner. RNA isolated from Brain3 was used for reverse transcription by a PrimeScript™ RT Reagent Kit (Takara, Japan) according to the manufacturer’s instructions. DNA was extracted from Muscle3. The primers were designed by the National Center for Biotechnology Information (NCBI) Primer–BLAST and synthesized by Thermo Fisher Scientific Inc. (Beijing, China) to amplify appropriate fragments for Sanger sequencing (Additional file 2: Table S1). The sites were considered verified if the cDNA sequence was heterozygous while the corresponding DNA sequencing was homozygous.

### Gene expression and editing level

To quantify the porcine gene expression level, Tophat2 [24] was applied with the default command options to align the RNA-seq reads against the reference genome (*Sus scrofa* 10.2.87). Then, HTseq [25] was used to count the reads aligned to each gene. Finally, fragments per kilobase million (FPKM), which is currently the most commonly used method for estimating gene expression levels [26], was calculated based on the length of the gene and the number of reads mapped to this gene. The RNA editing level at a given site was calculated as the ratio of reads supporting the edited base to the total number of reads covering the site. The editing level analysis was limited to RNA editing sites covered by at least 10 RNA reads.

### Functional enrichment analysis

We used the clusterProfiler package [27] to conduct a functional enrichment analysis based on Gene Ontology (GO) biological processes and Kyoto Encyclopedia of Genes and Genomes (KEGG) pathway terms. Human gene sets were chosen as the background. GO/KEGG terms with *q*-value < 0.05 were considered significantly enriched.

## Results

### Identification of RNA editing sites in swine

To accurately detect the candidate RNA editing sites at the transcriptome-wide level in swine, strand-specific poly(A)-positive RNA sequencing and matched DNA sequencing were performed using seven tissues from three pigs. After quality trimming, on average, 74.8 million reads were generated from each sample, with an average mapping rate of 85.6%. Approximately 88.1% of the 1338 million pass-filter reads obtained from the DNA sequencing were successfully aligned to the *Sus scrofa* reference genome. A summary of the deep sequencing process is provided in Table 1.

To fully utilize our sequencing data, the possible RNA editing events were detected with RES-Scanner, which requires matched RNA-seq and DNA-seq data to rule out genomic single nucleotide variants and automatically separates the plus-strand alignments from the minus-strand alignments for the strand-specific RNA-seq libraries to identify the correct genomic loci of origin. Using this method, in total, 163315 RNA editing events at 74863 sites were detected within our datasets (edited sites in different tissues or animals were considered separate events), including 68934 A-to-G editing sites (Additional file 3: Table S2).

### Validation of predicted RNA editing sites

**Fig. 1:**
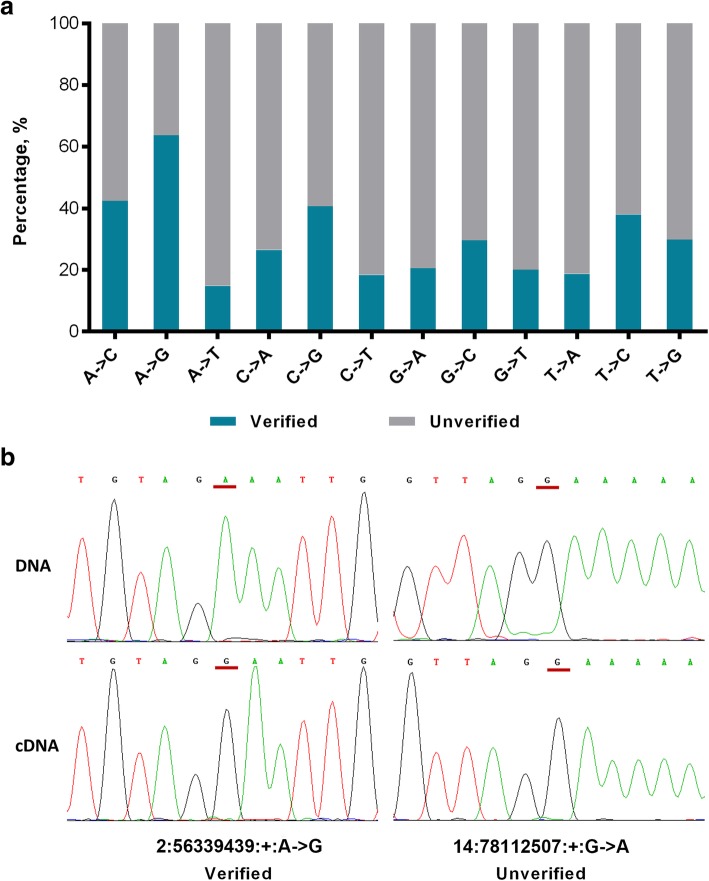
Verification of RNA editing sites. **a** The validated rate of each RNA editing type by EST BLAST searching. A verified editing site means that the site is supported by at least one edited EST sequence. **b** An example showing the genotyping results of the genomic DNA and RNA of one verified RNA editing site (Chr2:56339439:+:A- > G) and one unverified RNA-editing site (Chr14:78112507:+:G- > A) by Sanger sequencing. The sites are highlighted in red lines

First, we used an in silico approach as previously reported [28] to search for evidences of the detected RNA editing sites in porcine expressed sequence tags (ESTs) of NCBI. In brief, 50 bp upstream and 50 bp downstream of the flanking regions of the RNA editing sites were extracted and queried against the public pig ESTs using BLAST. Then, ESTs with an e-value < 10^− 5^ were counted. Of the 74863 editing sites, 67450 (90.1%) sites were covered by at least one EST sequence, and 45243 (60.4%) sites were found in at least one RNA edited EST clone. The validation rates significantly varied across the different RNA editing types as follows: 63.7% (43901/68934) of the A-to-G editing sites were supported by at least one RNA edited EST sequence, while less than 43% of the RNA editing sites were validated for each of the 11 non-A-to-G types (range from 14.9% to 42.5%) (Fig. 1a). Then, Sanger sequencing was carried out to experimentally validate 64 editing sites (47 A-to-G sites and 17 non-A-to-G sites) across five genomic feature regions comprising CDS, noncoding RNA (ncRNA) and untranslated, intronic and intergenic regions. Forty-three A-to-G sites (91.4%) and 10 other type sites (58.8%), including two C-to-A, three G-to-A and five T-to-C sites, were experimentally verified (Fig. 1b and Additional file 1: Figure S2). However, no known enzymes or biological mechanisms can catalyze the 10 non-A-to-G sites. Given these results, we focused on the A-to-G editing sites for further analysis.

### Characterization of porcine RNA editing sites

**Fig. 2:**
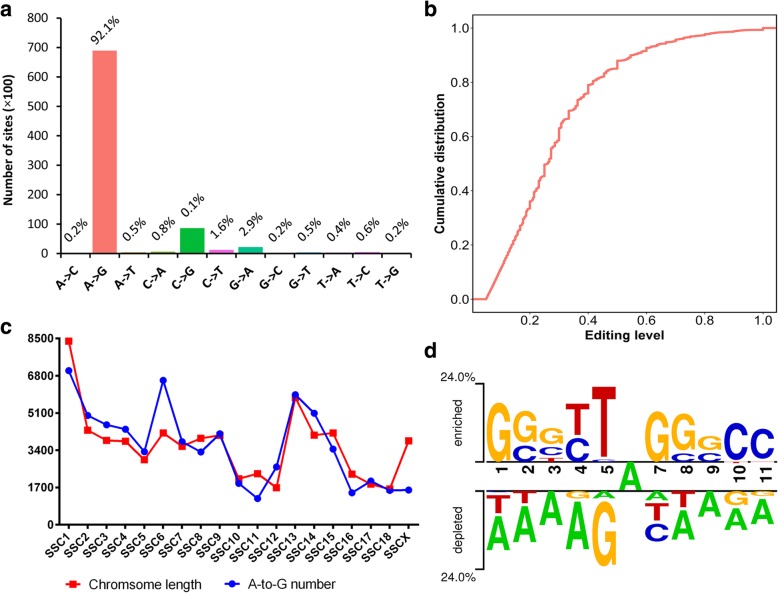
Characteristics of the pig editome. **a** Distribution of RNA editing types. **b** Cumulative percentage distribution of the editing levels of A-to-G sites. The editing level of a given editing site is determined by the number of reads with the edited base divided by the total reads. If the same site was detected in multiple samples, the highest editing level was used in the analysis. **c** Chromosome distribution of A-to-G sites. Chromosome length was normalized by multiplying by the number of editing sites/the total length of chromosomes. **d** Sequence preference of A-to-G RNA editing sites. The enriched (above the top line) and depleted (below the bottom line) nucleotides near the focal editing sites are displayed in Two-Sample Logo. The height of the letters depicts the level of preference/depletion

In this study, 12 types of RNA editing were detected, including all possible base substitutions as follows: A-to-G, A-to-C, A-to-T, C-to-A, C-to-G, C-to-T, G-to-A, G-to-C, G-to-T, T-to-A, T-to-C and T-to-G (Fig. 2a). Overall, the A-to-G substitution was the most common type, accounting for up to 92.1% of the identified RNA editing sites. The editing level of the A-to-G sites was low overall, and 90% of the detected A-to-G sites had editing levels less than 55% (Fig. 2b). We also observed that the identified A-to-G sites were widely and unevenly distributed across the *Sus scrofa* chromosomes (SSCs) as follows: more editing sites were detected on SSC1, SSC6 and SSC13 than on the other SSCs (Fig. 2c). The number of A-to-G sites had a similar tendency to change according to the length of the chromosomes. As expected, the number of RNA editing sites was significantly related to the chromosome length based on the correlation analysis (*r* = 0.82, *P* < 0.01). Then, we ranked the chromosomes according to the difference between the normalized chromosome length (multiplying by the number of editing sites/the total length of chromosomes) and observed RNA editing sites. The top four chromosomes were SSC6, SSCX, SSC1 and SSC11. Given the large number of RNA editing sites, we were able to determine whether there was a sequence preference in the vicinity of the detected A-to-G sites. Consistent with the known attributes of ADAR substrates, the nucleotide immediately upstream of the editing site showed significantly depleted G, while the downstream nucleotide favored G (Fig. 2d). In addition, an avoidance of A was observed in the 5′ and 3′ regions of the editing sites, which was also observed in the human inosinome [14] and rhesus macaque editome [5]. To explore whether porcine RNA editing sites also occurred in clusters similar to humans, the sites patterning in clusters ≥3 sites within 100 bp were calculated. We found that the extent of A-to-G site clustering widely ranged across the samples (from 24.8% to 98.7%, Additional file 4: Table S3). At the tissue level, the highest rate of A-to-G site clustering was found in the fat group (87.8% on average), while the lowest rate was found in the liver group (26.9% on average). A cross-species comparative analysis was performed to compare our detected editing sites and the human editome retrieved from the REDIportal (http://srv00.recas.ba.infn.it/atlas/) and DARNED (https://darned.ucc.ie/) databases. Using 50 bp flanking regions of the porcine RNA editing sites, BLAST analyses were performed against 50 bp flanking regions of the human sites. The sites supported by the ESTs with e-values *<* 0.001, identity *>* 85% and alignment length *>* 50 bp were considered conserved editing sites [28]. This analysis revealed 454 conserved A-to-G sites (Additional file 5: Table S4), which is comparable to a previous study [5].

### Analysis of the editing sites across genomic regions

**Fig. 3:**
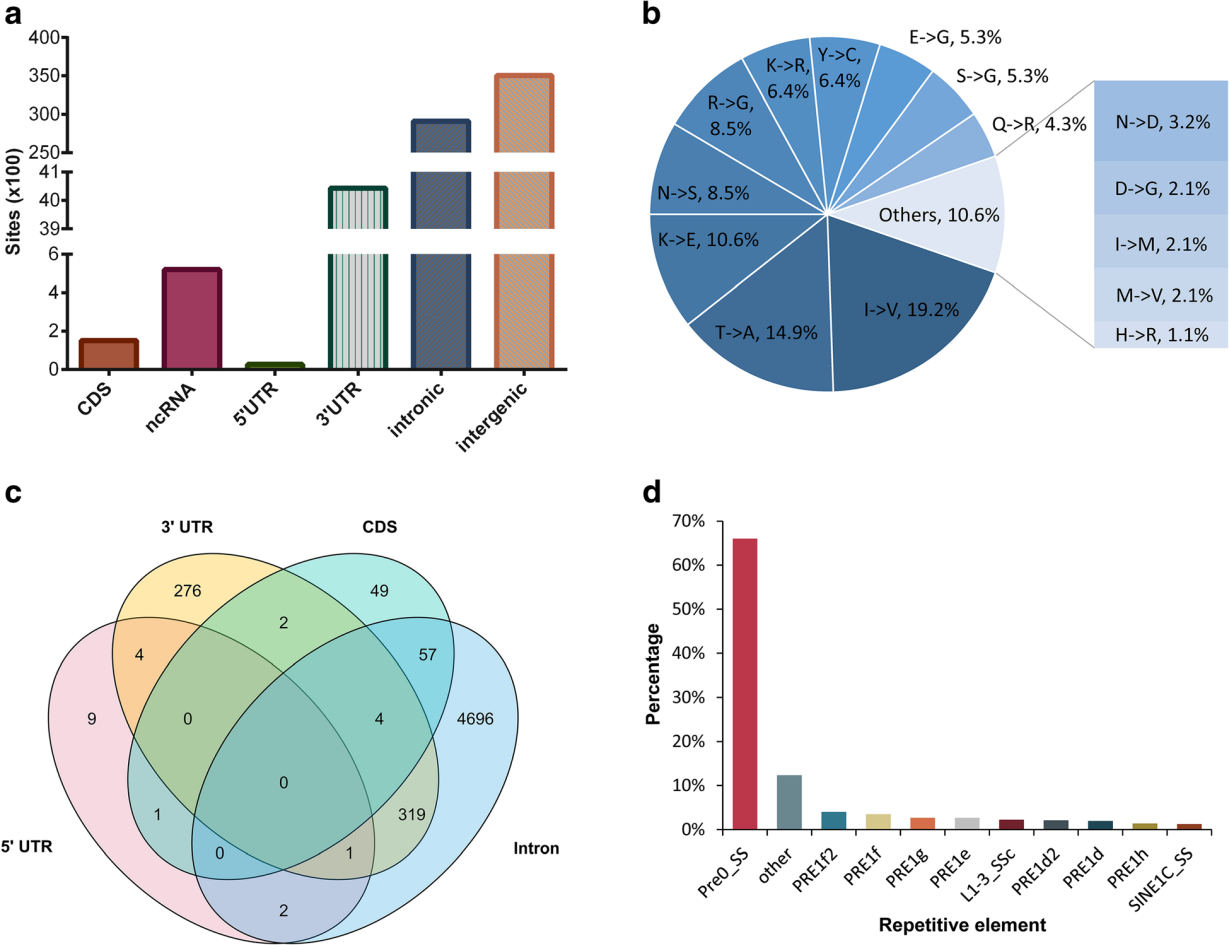
Signatures of editing sites in different genomic regions. **a** Statistics of A-to-G sites in different regions of genes. **b** Distribution of amino acid changes caused by missense editing. **c** Venn diagram displaying the distribution of A-to-G sites at the gene level. Most protein-coding genes undergo A-to-G editing in introns. **d** Distribution of A-to-G sites across repetitive elements. Approximately 66% of repetitive A-to-G sites fall to the Pre0_SS element, which is an active pig-specific SINE belonging to the PRE-1 family

Next, we studied the location characteristics of the detected RNA editing sites, and the priority was consistent with ANNOVAR [29]. Interestingly, the largest fraction of A-to-G sites was located in intergenic regions, followed by introns (Fig. 3a). In total, 520 A-to-G sites were located in ncRNA, and most (89.4%) of these sites occurred in introns. Less than 6% of the A-to-G sites were located in 3′ untranslated regions (UTR), including 592 sites that overlapped with the target sites of miRNA seed regions (2^nd^ to 8^th^ nucleotides, the key region involved in the recognition between a miRNA and the 3´UTR of its target mRNA [30]) predicted by miRanda [31]. The top 20 miRNAs according to the number of edited targets are shown in Additional file 6: Table S5. Furthermore, we found that 341 of the remaining 3451 3´UTR sites potentially generated novel miRNA targets. In addition, 151 A-to-G sites were detected in CDS, including 94 sites that could lead to nonsynonymous amino acid changes (Additional file 7: Table S6). Among the 94 missense sites, 30 sites had editing evidence in all seven studied tissues, and 59 sites were detected in at least two tissues. Notably, the top three most frequent substitution types, i.e., isoleucine to valine (I-to-V), threonine to alanine (T-to-A) and lysine to glutamate (K-to-E), accounted for more than 44% of all amino acid conversions (Fig. 3b). Up to 70.2% of the missense A-to-G sites were observed at the first or second position in codons. The putative impacts of the amino acid replacements predicted by snpEff [32] demonstrated that all 94 missense variances were likely to be moderate. Only four missense sites (Chr4:98044799, Chr7:10-2789222, 8:48244993 and Chr11:22178068) have been previously identified in a single female pig from an F_2_ population [22]. This finding might be caused by a difference in breeds.

While concentrating on the distribution of A-to-G sites within a gene model, we found that 92.8% of the 5420 protein-coding genes undergoing RNA editing were edited in only one genic region (Fig. 3c). While 93.7% of the edited genes had intronic editing sites, only 7.1% of the edited genes simultaneously had intronic and other genic region editing sites. The considerable intronic RNA editing sites and the observation that most genes were edited in intronic regions suggested that A-to-G editing may impact splicing as previously reported [33]. It is universally acknowledged that in primates, RNA editing sites are mostly located in Alu elements, which are categorized into short interspersed nuclear elements (SINEs). This property allowed us to understand the association between the RNA editome and repetitive elements in the pig. AnnotateTable.py from REDItools [34] was used to determine which repetitive elements contained the identified RNA editing sites. Consistent with previous studies [5, 22, 35], 97.3% of the RNA editing sites located in the repeats were A-to-G conversions (Additional file 1: Figure S3), and 94.1% of the repetitive A-to-G sites were located in SINEs (Additional file 1: Figure S4). By further subdividing the repetitive element families, we found that 88.6% of the repetitive A-to-G sites occurred within the PRE-1 family, and, notably, approximately 66% of these sites occurred in the Pre0_SS element, which is an active pig-specific SINE belonging to the PRE-1 family (Fig. 3d).

### Distribution of RNA editing sites across porcine tissues

**Fig. 4:**
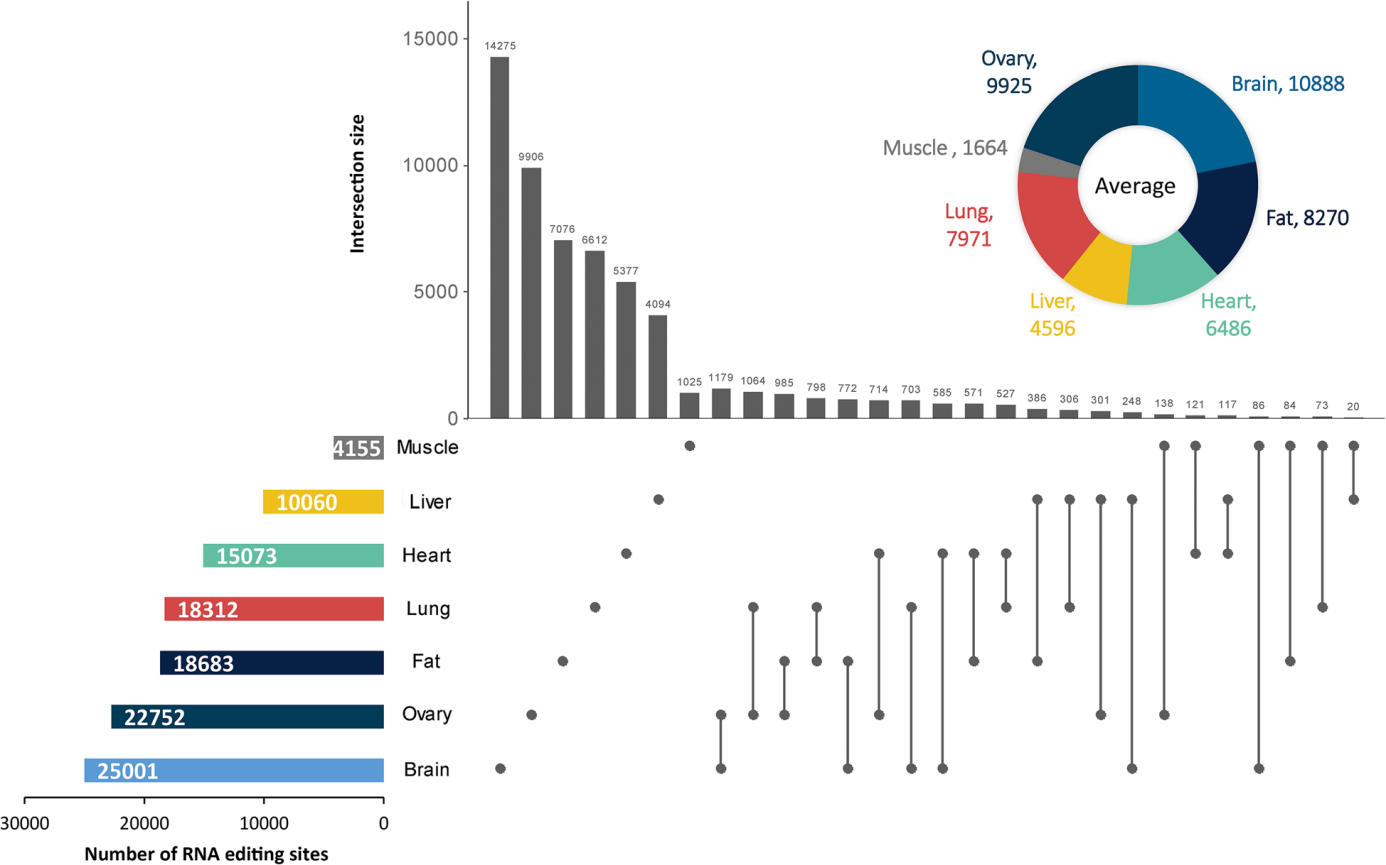
Landscape of RNA editing sites across porcine tissues. The doughnut chart displays the average number of A-to-G sites in each tissue group. The horizontal bar chart displays the nonredundant A-to-G sites in each tissue group. The vertical bar chart shows the number of shared RNA editing sites across tissues

**Fig. 5:**
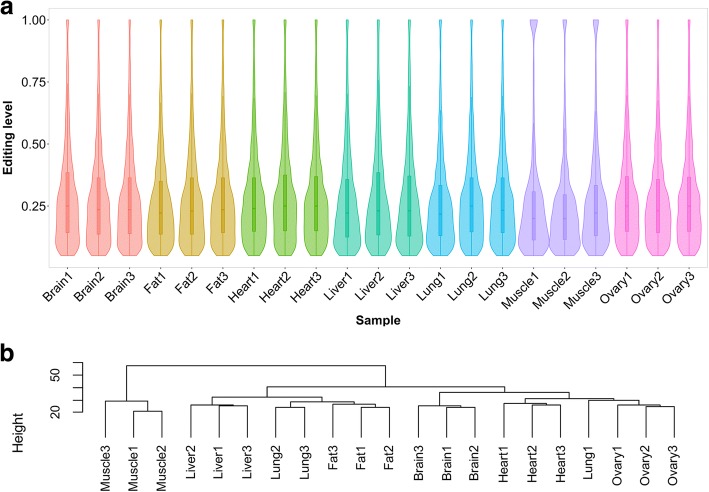
Statistical features of RNA editing levels within and across samples. **a** The distribution of RNA editing levels across samples. Overall, the RNA editing levels are similar across tissues and within each tissue group. **b** Hierarchical clustering of RNA editing levels at all A-to-G sites across multiple tissues and individuals

The number of A-to-G sites greatly varied across tissues and pigs (Fig. 4 and Additional file 1: Figure S5). Only 513 RNA editing sites spanning 169 expressed genes were shared by the seven tissues (Additional file 8: Table S7). Less than 10% of the RNA editing sites were shared across the three samples of each tissue. These observations highlighted the variety and diversity of co/posttranscriptional modification. Overall, the brain was the most edited tissue, with an average of 10888 A-to-G sites, followed by the ovary, and the muscle had the least number of A-to-G sites (on average 1664). After removing the duplicates, the number of nonredundant RNA editing sites in the tissues ranged from 4155 to 25001. The large number of sites in each tissue suggested that RNA editing is likely functionally important in nonbrain tissues. Unexpectedly, although an obvious difference was discovered in the number of A-to-G sites per sample, the detected sites exhibited strong comparability in editing levels, with the median level ranging from 0.2 to 0.25 (Fig. 5a). According to the hierarchical clustering analysis, we further found that the interindividual variations of the editing levels were smaller than the cross-tissue variation (Fig. 5b).

**Fig. 6:**
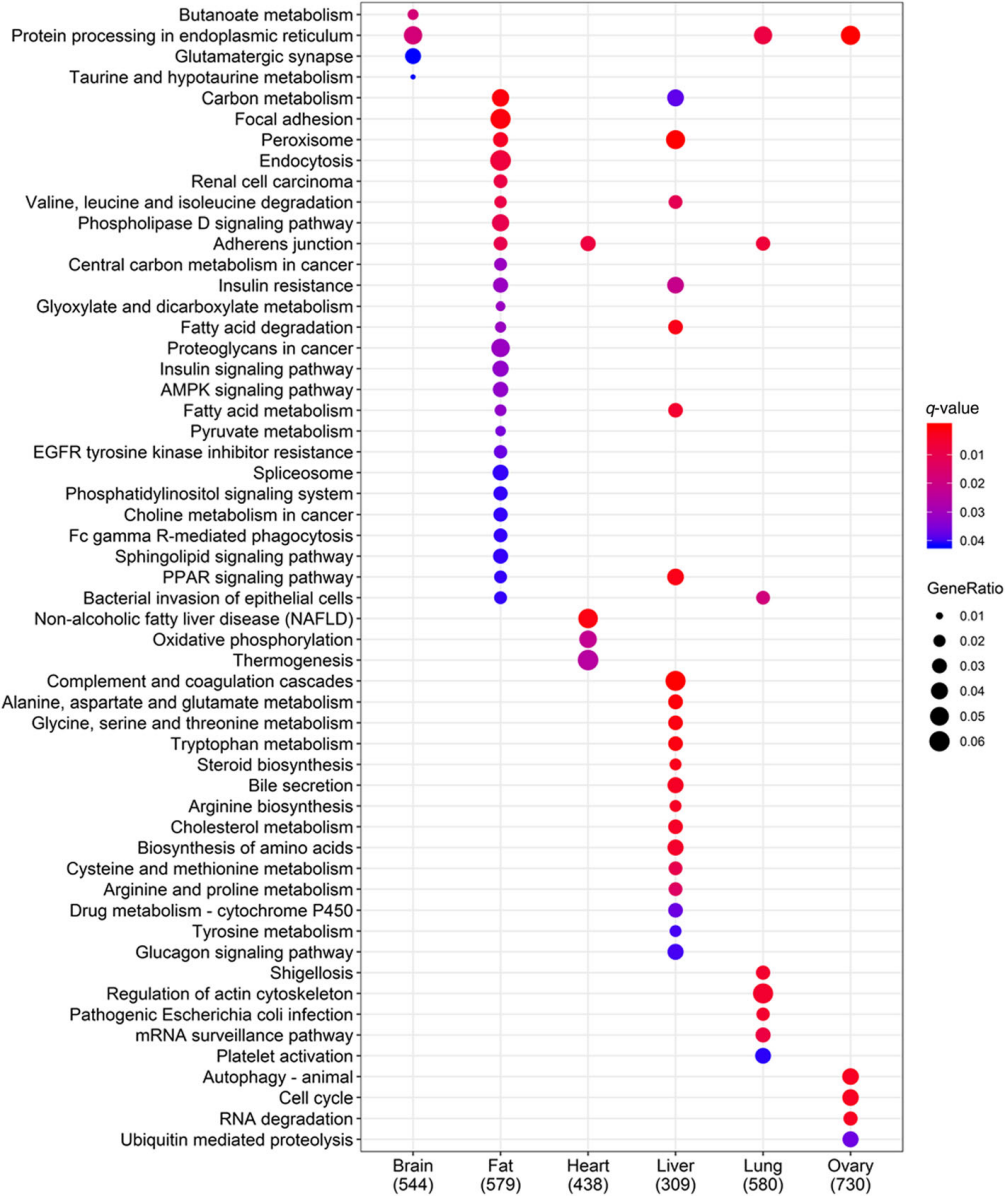
Pathway enrichment analysis of the genes containing tissue-specific RNA editing. Dot plot of the enriched KEGG pathways in each tissue. Dot color indicates the statistical significance of the enrichment (*q*-value); dot size represents the fraction of genes annotated to each term

Interestingly, most tissue-shared sites were present in noncoding regions, especially the 3´UTR, and less than 6% of the shared sites were located in CDS. This result suggested that common RNA editing may function by regulating the expression of specific genes. While concentrating on the tissue specificity of RNA editing, we found that a host of A-to-G sites occurred only in specific tissues (Fig. 4). This finding highlighted the strong tissue specificity of RNA editing. Therefore, we further counted the tissue-specific sites by referring to a previously reported study [14]. Briefly, we mapped all detected A-to-G sites on the expressed genes with an FPKM > 1 and then selected tissue-specific sites from the sites in the expressed genes. Unexpectedly, the ovary, rather than the brain, had the largest number of tissue-specific editing sites (Additional file 9: Table S8). Notably, less than 10% of the tissue-specific editing sites occurred in tissue-specific expressed genes within each tissue type. To characterize the functional significance of the tissue-specific editomes, a functional enrichment analysis of the genes with tissue-specific editing sites was carried out using the Bioconductor package clusterProfiler. As expected, these genes were significantly enriched in biological processes related to their respective tissue functions, such as “dendrite development” in the brain (*q*-value = 9.07 × 10^−5^), “lipid modification” in the fat (*q*-value = 4.98 × 10^−4^), “cardiac muscle cell differentiation” in the heart (*q*-value = 1.07 × 10^−3^), “carboxylic acid catabolic process” in the liver (*q*-value = 3.68 × 10^−14^), “vesicle organization” in the lung (*q-*value = 6.21 × 10^−3^), “actomyosin structure organization” in the muscle (*q*-value = 3.28 × 10^−2^), and “cell cycle G2/M phase transition” in the ovary (*q*-value = 2.93 × 10^−8^). The top 5 GO terms of each tissue according to the *q*-values are displayed in Additional file 1: Figure S6. Based on the pathway analysis (Fig. 6), we discovered obviously different enriched pathways among these tissues. In addition, most pathways enriched in each tissue were related to their respective tissue function, such as “glutamatergic synapse” in the brain (*q*-value = 4.33 × 10^−2^), “AMPK signaling pathway” in the fat (*q*-value = 3.64 × 10^−2^), and “fatty acid metabolism” (*q*-value = 1.21 × 10^−3^) in the liver. Specifically, more pathways were enriched in the fat and liver than in the other tissues, while no pathway was enriched in the muscle. Similar results were obtained even after excluding the tissue-specific expressed genes (Additional file 1: Figure S7).

### Association between *ADAR* expression levels and the porcine RNA editome

**Fig. 7:**
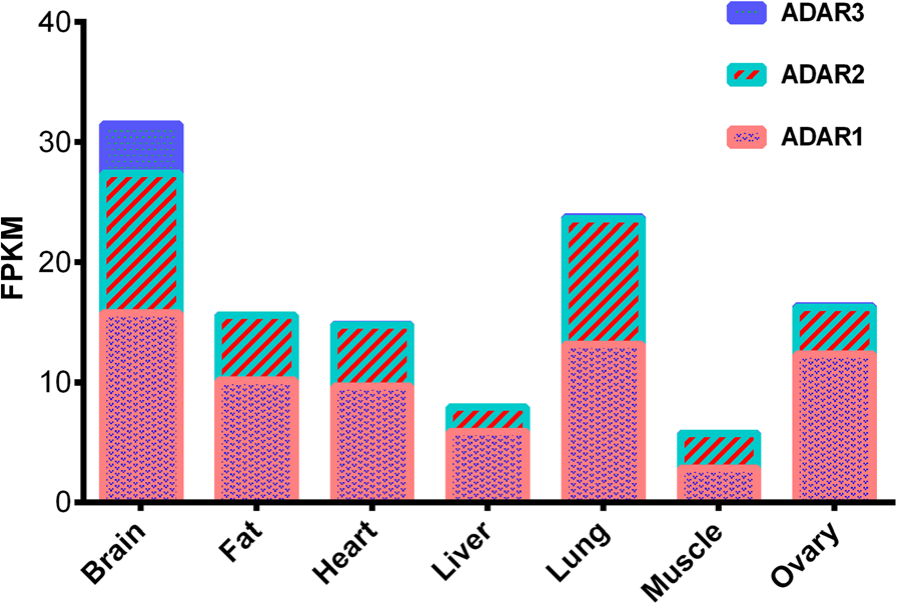
Expression levels of ADAR genes across porcine tissues

It is well known that A-to-G editing is catalyzed by ADAR enzymes. Hence, we investigated whether the tissue differences in RNA editing are related to the differential expression of the *ADAR* genes. First, we calculated the expression levels of the *ADAR1*, *ADAR2* and *ADAR3* genes using our RNA-seq data. The total expression of the *ADAR*s is the highest in the brain, and the lowest expression is in the muscle (Fig. 7). We also found that *ADAR3* was nearly exclusively expressed in the brain. Next, we estimated the correlations between the expression of the *ADARs* and the RNA editing site number and between the expression of the *ADARs* (except for *ADAR3,* which is only expressed in the brain) and the global RNA editing level (summing the editing levels at all positions) at the sample level. This analysis revealed a strong and statistically significant correlation between both the number of editing sites (*r* = 0.89, *P <* 0.01) and the global level of editing (*r* = 0.89, *P* < 0.01) with the expression of *ADAR1* (Additional file 1: Figure S8). Statistically significant correlations were also observed for *ADAR2*, but these correlations were not strong (*r* = 0.59 for RNA editing number; *r* = 0.58 for global RNA editing level). Then, by repeating this analysis at the tissue group level, statistically significant correlations were found only for *ADAR1* (number of editing sites: *r* = 0.96, *P* < 0.01; global level of editing: *r* = 0.95, *P* < 0.01) (Additional file 1: Figure S9). These observations indicated that *ADAR1* may be the primary editor of the A-to-G sites.

## Discussion

To accurately call the RNA editing sites, we meticulously designed our experiment. First, long paired-end reads were used to improve the genome mappability and facilitate the identification of hyperedited reads [36]. Second, strand-specific sequencing protocols were used to identify the correct genomic loci of origin while significantly controlling for potentially ambiguous calls due to widespread anti-sense expression [5, 37]. Then, matched RNA and DNA sequencing was performed in porcine tissues from the same individuals, which efficiently eliminated genetic variations compared with working with RNA-seq data alone. Finally, RES-Scanner, which is an all-in-one tool that incorporates sophisticated statistical models, was applied to effectively distinguish the real RNA editing sites from potential false positives. These efforts ensured the accuracy and quantity of the editing site identification.

Overall, we identified 74863 editing sites, which is far less than that detected in a similar survey in humans (2013010 sites) [14]. A certain proportion of this difference could be attributed to the primate-specific Alu elements, which are active SINE retrotransposons. We further noted that there are more editing sites in pigs than in cows [28] and chickens [38]. The PRE-1 elements are pig-specific SINE retrotransposons that possess properties similar to Alu [39], and a previous study reported that RNA editing in swine is associated with PRE-1 elements [22]. Similar to the previous study, our results showed that 88.6% of the 67136 repetitive A-to-G sites occurred within the PRE-1 family. Hence, PRE-1 retrotransposons may contribute to the difference in the number of RNA editing sites between pigs and humans or other animals.

Consistent with previous studies in primates [5, 6, 40], > 90% of the identified sites are of the A-to-G type, which is higher than that reported in the pig in a previous study (75% A-to-G changes) [22]. This indicates the high accuracy of our detection referring to the study of Porath et al. [41]. Approximately 64% of the A-to-G sites were validated by at least one EST sequence, which is consistent with studies in mice (> 55% of validation) [16] and cows (66% of validation) [28]. To understand the mechanism of target recognition, the sequence contexts of the A-to-G editing sites were analyzed. The sequence preference in pigs is similar to that in humans [42], mice [43], cows [28] and chickens [38], indicating the conversion of the recognition mechanism. It is well known that A-to-G sites can be grouped into clusters and that an editing cluster increases the reliability of the contained editing sites. In the present study, we found strong tissue specificity of A-to-G site clustering.

In our research, the intergenic regions contained the largest number of RNA editing sites, which is different from humans, where intronic editing sites are the most common. A possible explanation for the difference is that the porcine genome annotation is of poor quality compared with the human annotation, and many unannotated genes are present (25322 pig genes vs 63305 human genes in Ensembl release 87). However, the abundance of RNA editing within the intronic and intergenic regions is unexpected because our RNA-seq libraries were purified using oligo(dT) beads to enrich polyadenylated mRNAs that had undergone splicing. This finding could be partially attributed to intron retention [44]. Moreover, SINEs may be another reason for this unexpected observation. SINEs are sequences of noncoding DNA that generally have more or less degenerate poly(A) tails [45]. It is entirely possible that a standard oligo(dT) protocol introduces SINEs into RNA-seq libraries, increasing the reads that overlap with intronic and intergenic sequencing. We found 94 nonsynonymous A-to-G editing sites resulting in 15 amino acid change classes across 76 genes. Notably, a number of RNA editing sites that control I/V, Y/C and Q/R in *GRIK2,* I/V in *COPA,* I/V in *KCNA1,* T/A in *ACOT4,* R/G in *GRIA2,* I/V, I/M and N/S in *HTR2C* and R/G in *GRIA3* have been identified in pigs in other studies [22, 46]. The three RNA editing sites in *GRIK2* are considered important for regulating calcium permeability [47]. For *COPA,* I/V editing was detected in the current seven tissues, and its hypoediting is associated with hepatocellular carcinoma pathogenesis in humans [48]. The I/V recoding of *KCNA1* via RNA editing affects the action potential shape, signal propagation and the firing pattern by accelerating the KCNA1 channel recovering from inactivation [49]. Consistent with previous work [22], *ACOT4* was also found to be edited in fat, but the function of its editing is unknown. The R/G recoding in *GRIA2* and *GRIA3* leads to faster desensitization recovery [50]. The RNA editing in *HTR2C* can reduce the efficacy of the interaction between receptors and their G protein [51]. In addition to the above reported genes, we discovered several genes with nonsynonymous A-to-G editing sites for further studies investigating the function of RNA editing.

Although obvious variation was observed across the editing profiles of the samples, the intrapopulation variability in the editing levels is lower than that across tissues, suggesting a similarity to gene expression regulation [52]. By comparing the A-to-G editing profiles among seven porcine tissues, we discovered that most A-to-G sites were tissue-specific. However, 39.9% of the hepatic A-to-G sites were common to adipose, which is comparable to previously reported findings [22]. In the functional enrichment study, we found that the tissue-specific editing site-containing genes were significantly enriched in pathways related to their respective tissue functions and that the enriched pathways obviously differed among these tissues. The glutamatergic synapse pathway is a major excitatory neurotransmission pathway in the mammalian central nervous system [53]. The AMPK signaling pathway can regulate adipose lipolysis and fat oxidation [54]. In fat, AMPK could directly phosphorylate lipases, such as hormone sensitive lipase and adipocyte triglyceride lipase [55, 56]. The liver plays an important role in fatty acid metabolism [57]. These observations demonstrate that RNA editing may play an important role in porcine tissue regulation. Moreover, there were more enriched pathways in the fat and liver than in the other tissues. However, no pathway showed significant enrichment in the muscle. Consistent with this study, in mammals, fewer RNA editing sites have been reported in muscle than in other tissues [5, 14]. In our study, we detected only 547 muscle-specific RNA editing sites that occurred in 410 expressed genes (FPKM > 1). Of the 410 genes, 149 genes were mapped to KEGG pathways. Hence, the fewer genes compared to the gene numbers in other tissues (310~732 genes) might contribute to the observation that no pathway was enriched in the muscle. Our findings might provide a new layer of regulation underlying complex traits in pigs. Furthermore, RNA edits provide information that has been unexplored at the DNA level. Hence, RNA edits could be integrated with SNPs and used in genome selection to improve the accuracy of breeding. In addition, given that RNA editing is dynamically regulated, RNA editing sites could be used as markers to monitor development, health and response to feed in breeding.

The ADAR enzymes have been shown to be essential for normal life and development in mice [8, 9]. Consistent with other mammals, three ADARs exist in pigs, including ADAR1, ADAR2 and ADAR3. Only ADAR1 and ADAR2 have been shown to be enzymatically active [58]. However, ADAR3 can inhibit RNA editing by competitively binding double stranded RNA [59]. Consistent with previous studies, *ADAR1* and *ADAR2* were highly expressed in the brain and lung, and *ADAR3* was exclusively expressed in the brain [5, 14]. Although the sample distribution of the A-to-G sites was significantly and positively correlated with the expression of the *ADARs*, the tendency to change did not perfectly align. This observation shows that the editing enzymes play an important role in RNA editing regulation but cannot explain all modification.

## Conclusions

This study identified 74863 RNA editing sites using matched RNA and DNA sequencing data and revealed the comprehensive profile of RNA editing in pigs. We also provide further evidence supporting a previous observation that pig transcriptomes are highly editable in PRE-1 SINE elements. Furthermore, the functional analysis of the genes with tissue-specific editing sites in each tissue revealed the potentially functional importance of RNA editing in porcine tissue regulation. Our study largely extends the list of RNA editing sites in swine and provides deeper insight into the characteristics of the pig editome.

## Additional files

Additional file 1: **Figure S1.** Overview of the experimental design. **Figure S2.** Results of the Sanger sequencing validation of all 64 selected editing sites. For each candidate editing site (indicated by genome coordinates and red arrow), raw chromatograms of sequences derived from cDNA and matched DNA samples are shown. Unverified sites are marked with an asterisk. **Figure S3.** Distribution of RNA editing types in repetitive sequences. **Figure S4.** Distribution of A-to-G sites across major repeat families. **Figure S5.** Overlap of RNA editing sites among the three samples of each tissue. **Figure S6.** Dot plot of the top five enriched GO biological process terms in each tissue. Dot color indicates statistical significance of the enrichment (*q*-value); dot size represents the fraction of genes annotated to each term. **Figure S7.** Dot plot of the enriched KEGG pathways after removing the tissue-specific expressed genes. Dot color indicates the statistical significance of the enrichment (*q*-value); dot size represents the fraction of genes annotated to each term. **Figure S8.** RNA editing vs expression of *ADAR*s in all samples. a) We correlated the number of A-to-G sites and *ADAR1* expression levels in all samples (*n* = 21). b) We correlated the number of A-to-G sites and *ADAR2* expression levels in all samples (*n* = 21). c) and d) We calculated the correlations between the overall RNA editing levels and the expression values of *ADAR1* and *ADAR2* in all samples (*n* = 21). Correlation coefficients (r) and *P*-values are shown in each graph. **Figure S9.** RNA editing vs the expression of *ADAR*s in tissue groups. a) We correlated the number of A-to-G sites and *ADAR1* expression levels in all tissue groups (*n* = 7). b) We correlated the number of A-to-G sites and *ADAR2* expression levels in all tissue groups (*n* = 7). c) and d) We calculated the correlations between the overall RNA editing levels and the expression values of *ADAR1* and *ADAR2* in tissue groups (*n* = 7). Correlation coefficients (r) and *P*-values are displayed in each graph. (PDF 87057 kb)

Additional file 2: **Table S1.** Primers used in the Sanger sequencing. (XLSX 135 kb)

Additional file 3: **Table S2.** RNA editing sites identified in this study. (XLSX 6798 kb)

Additional file 4: **Table S3.** Clustering of A-to-G sites in each sample. (XLSX 9 kb)

Additional file 5: **Table S4.** Conserved A-to-G editing sites between pigs and humans. (XLSX 25 kb)

Additional file 6: **Table S5.** Top 20 miRNAs according to the number of edited targets. (XLSX 9 kb)

Additional file 7: **Table S6.** A-to-G sites resulting in amino acid changes. (XLSX 13 kb)

Additional file 8 **Table S7.** RNA editing sites shared by the seven examined tissues. (XLSX 27 kb)

Additional file 9 **Table S8.** Statistics of the tissue-specific A-to-G editing sites. (XLSX 9 kb)

## Abbreviations

ADARs: Adenosine deaminases acting on RNA
A-to-G: Adenosine-to-guanosine
CDS: Coding regions
EST: Expressed sequence tag
FPKM: Fragments per kilobase million
GO: Gene Ontology
KEGG: Kyoto Encyclopedia of Genes and Genomes
NCBI: National Center for Biotechnology Information
ncRNA: noncoding RNA
SINE: Short interspersed nuclear element
SSC: *Sus scrofa* chromosome
UTR: Untranslated regions
