# Supplementary figures and images for: Genome-wide identification of RNA editing in seven porcine tissues by matched DNA and RNA high-throughput sequencing
Source: J Anim Sci Biotechnol. 2019 Mar 13;10:24. doi: 10.1186/s40104-019-0326-9 (PMC6415349; doi:10.1186/s40104-019-0326-9)

Figure S1

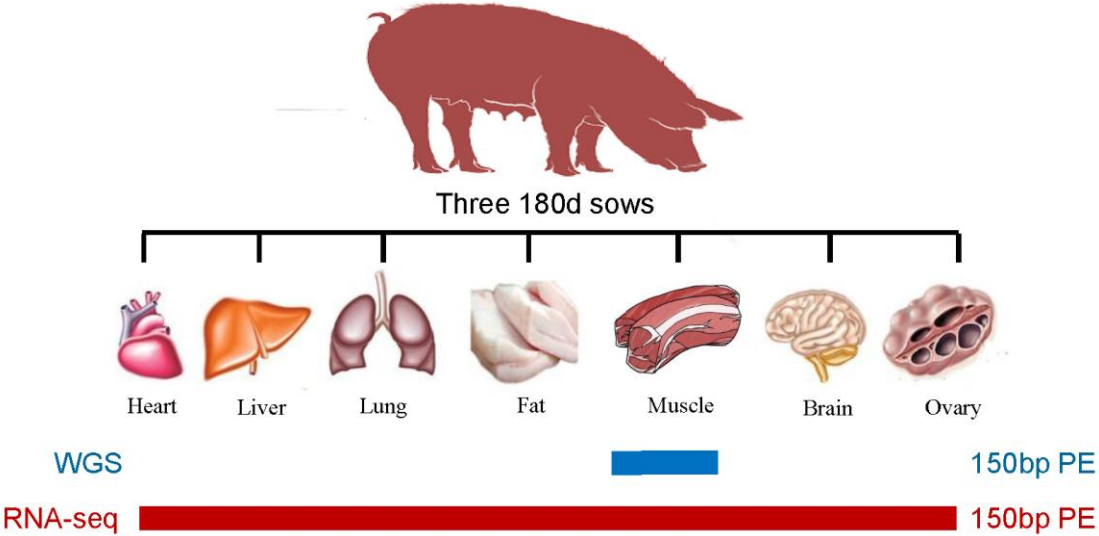

Figure S2  
S2-1

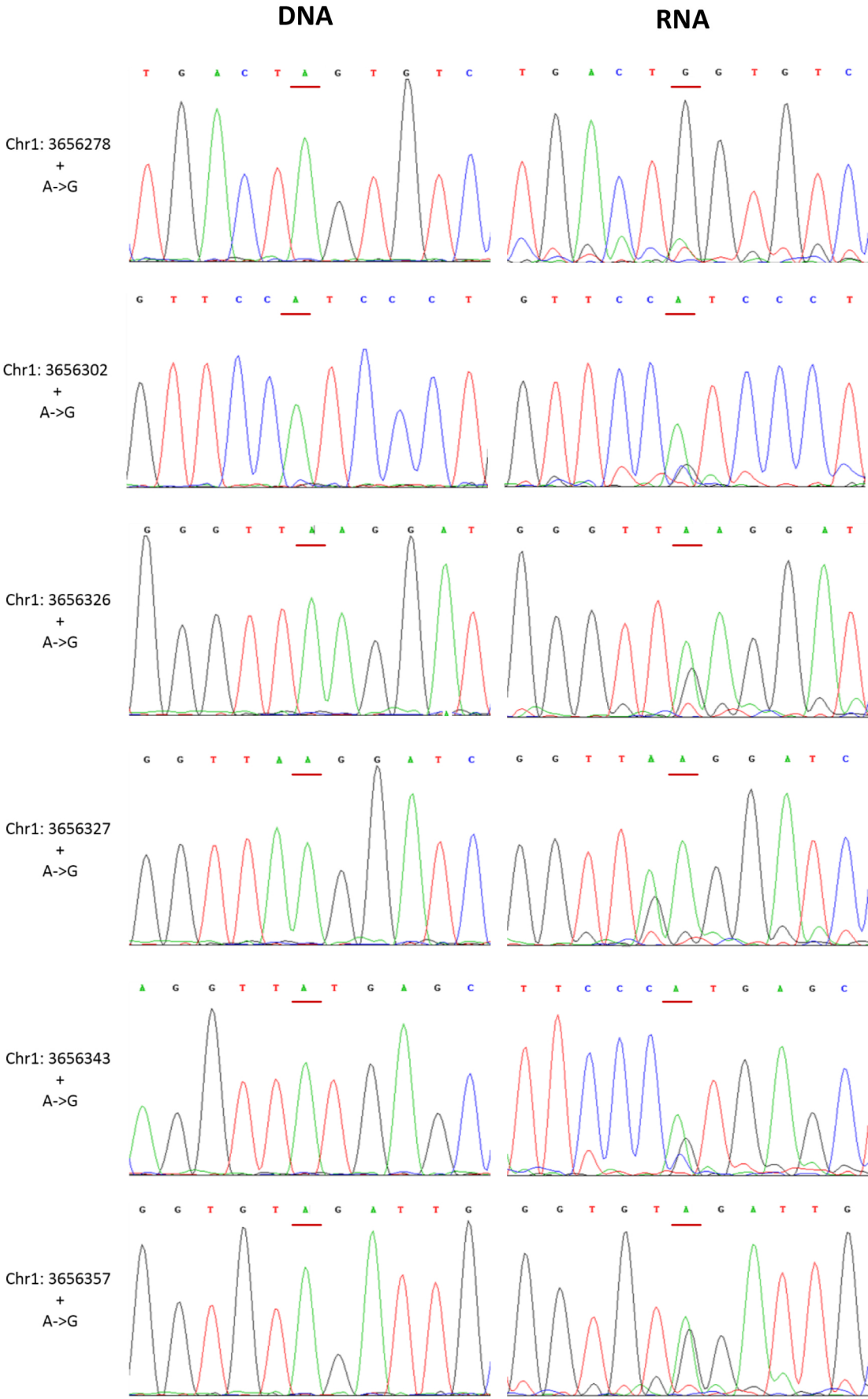

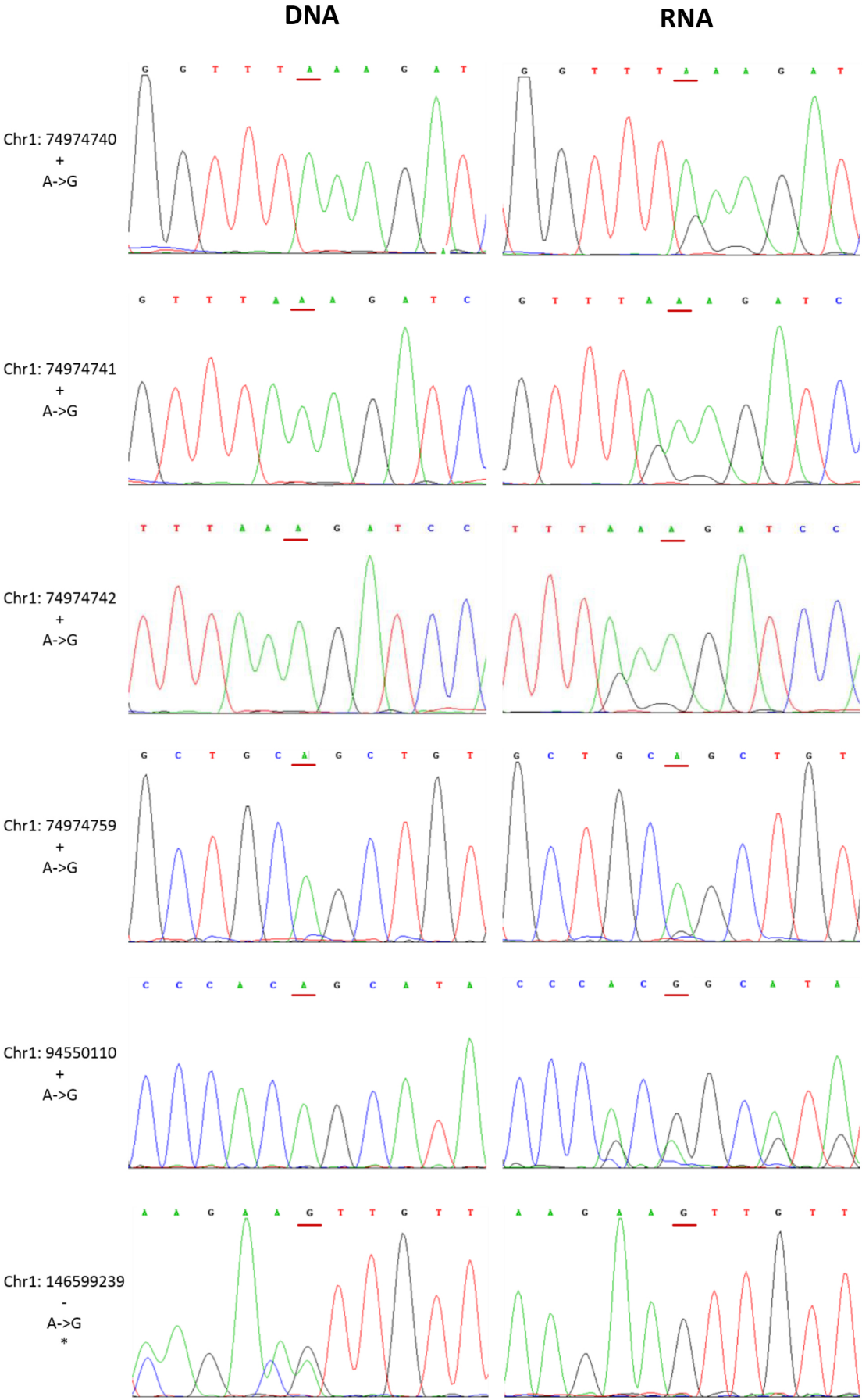

S2-3

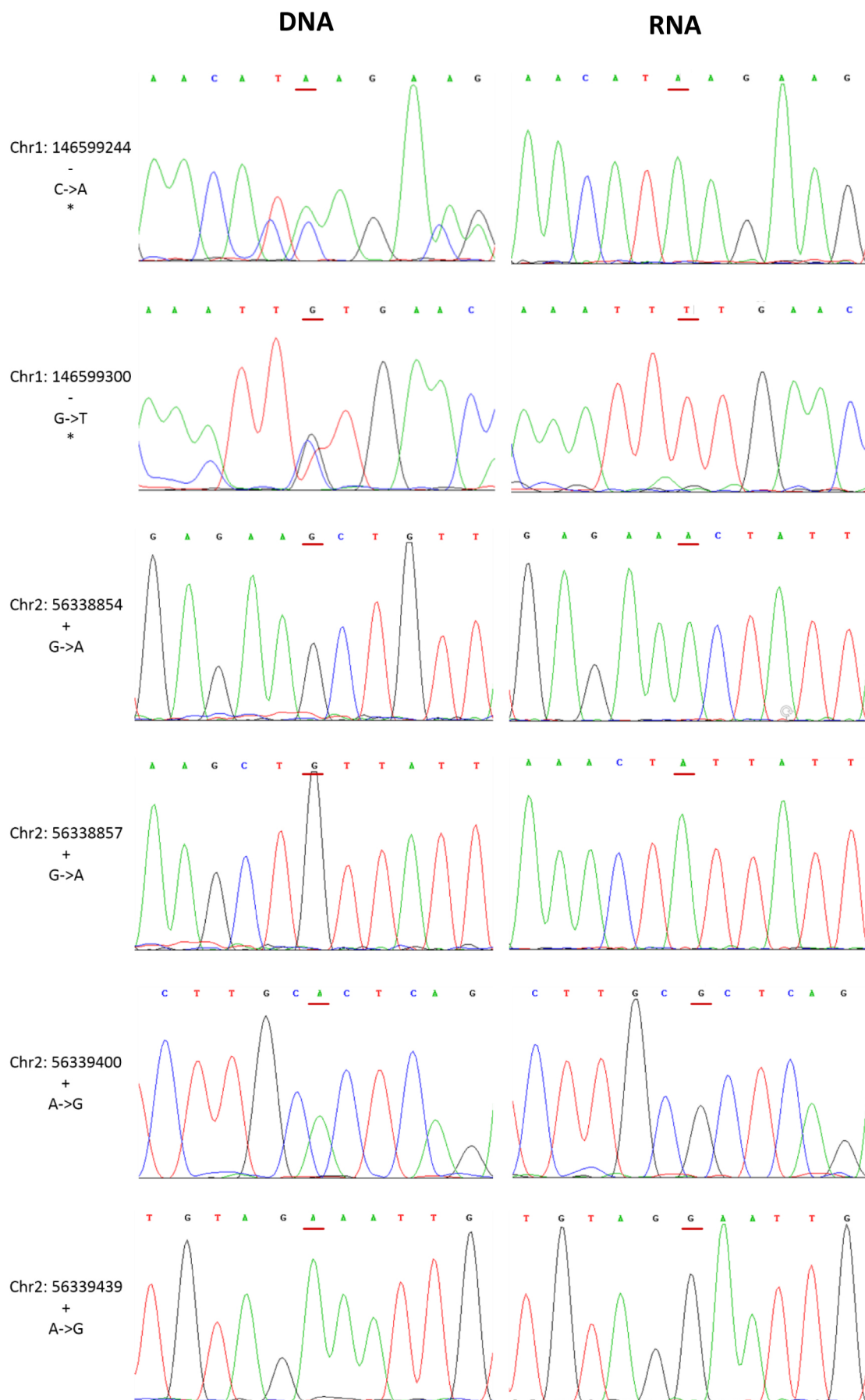

S2-4

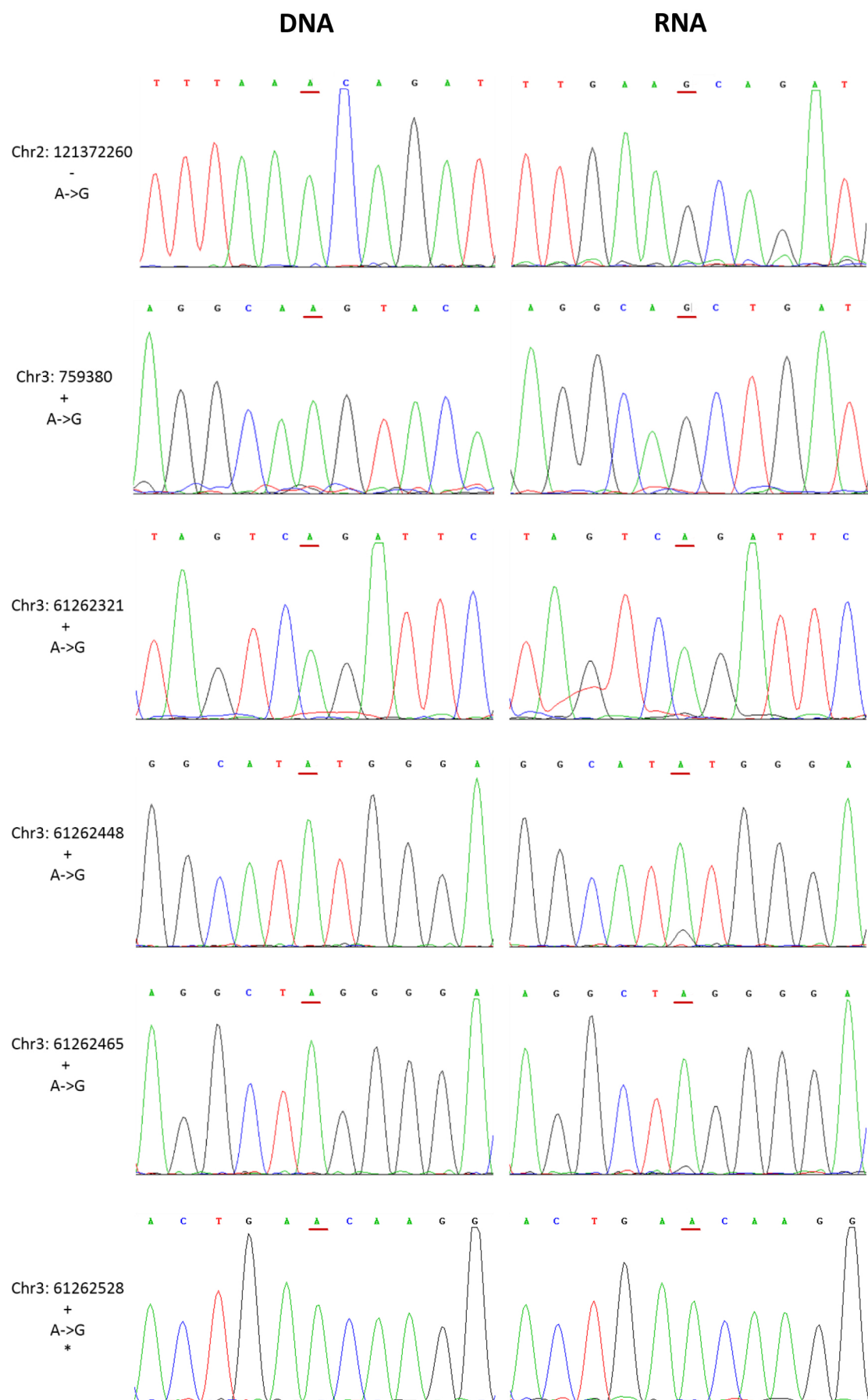

S2-5

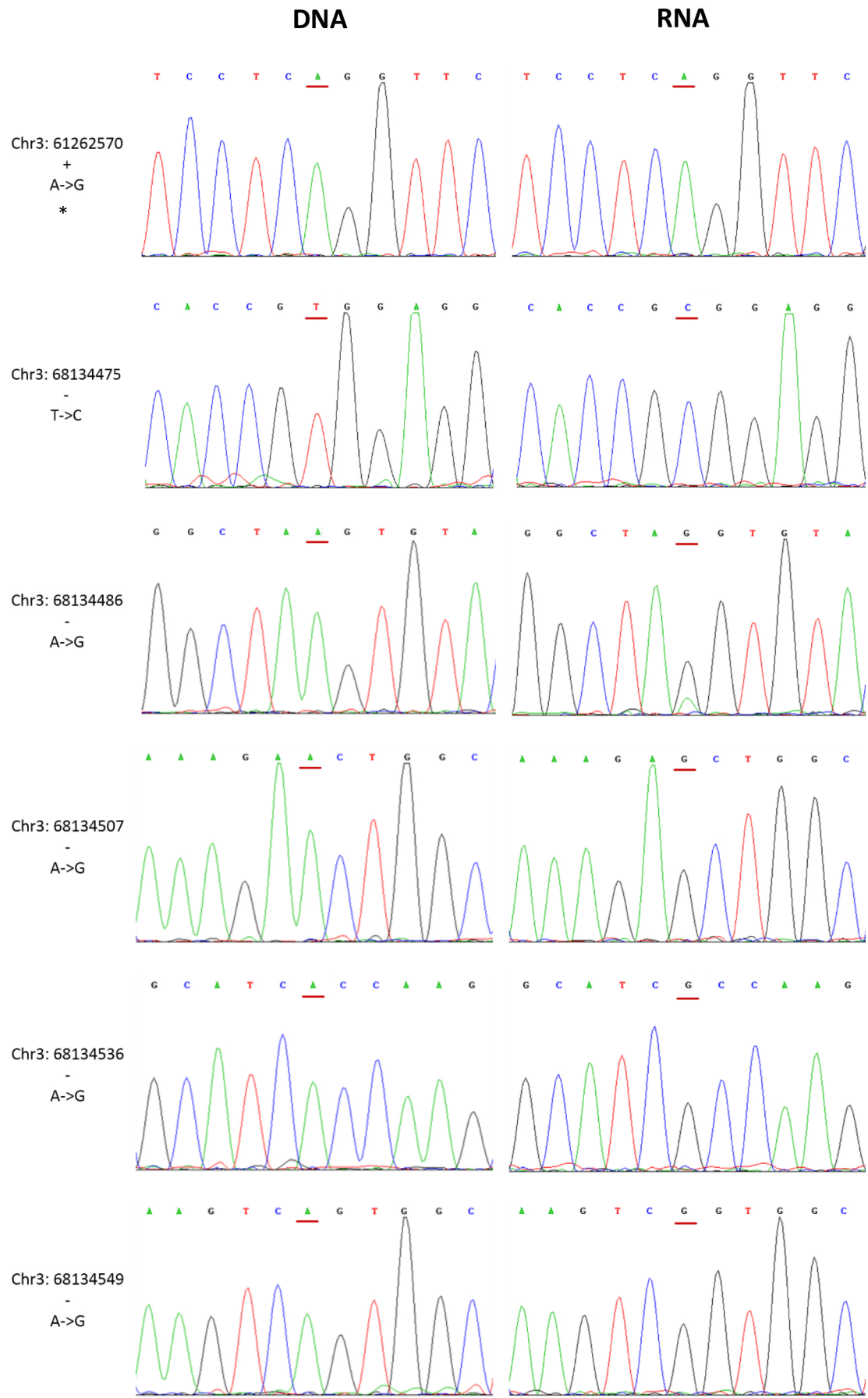

S2-6

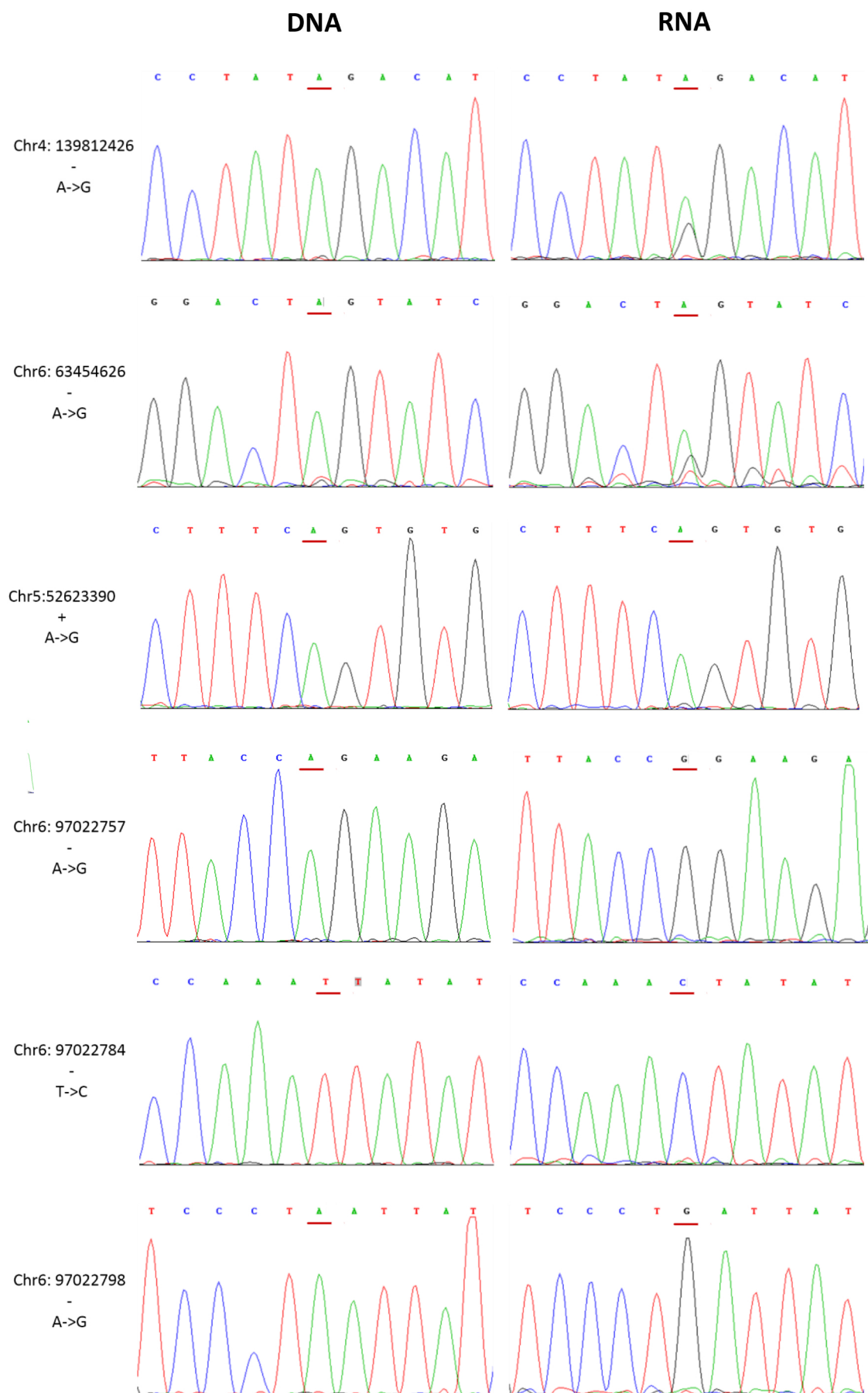

S2-7

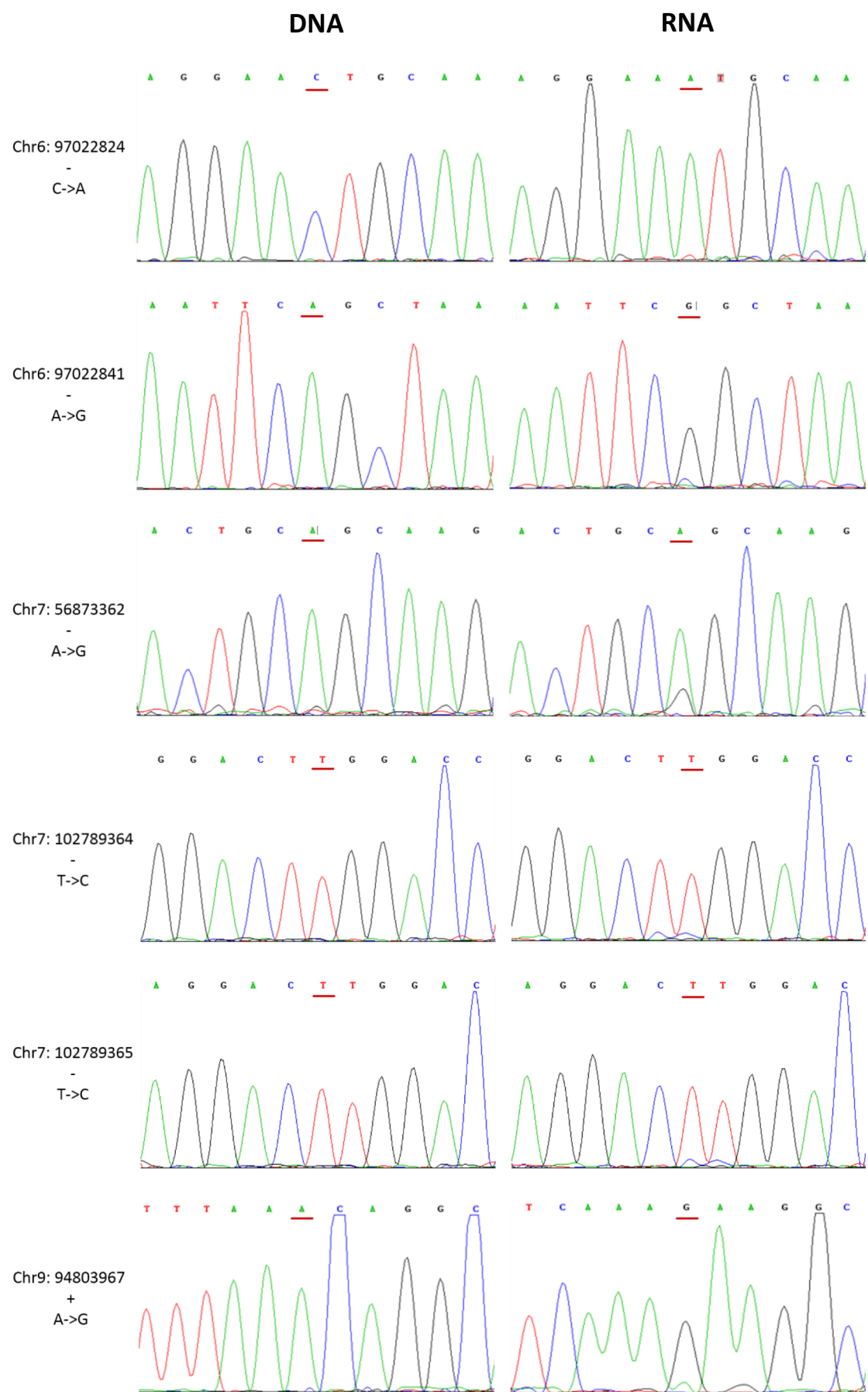

S2-8

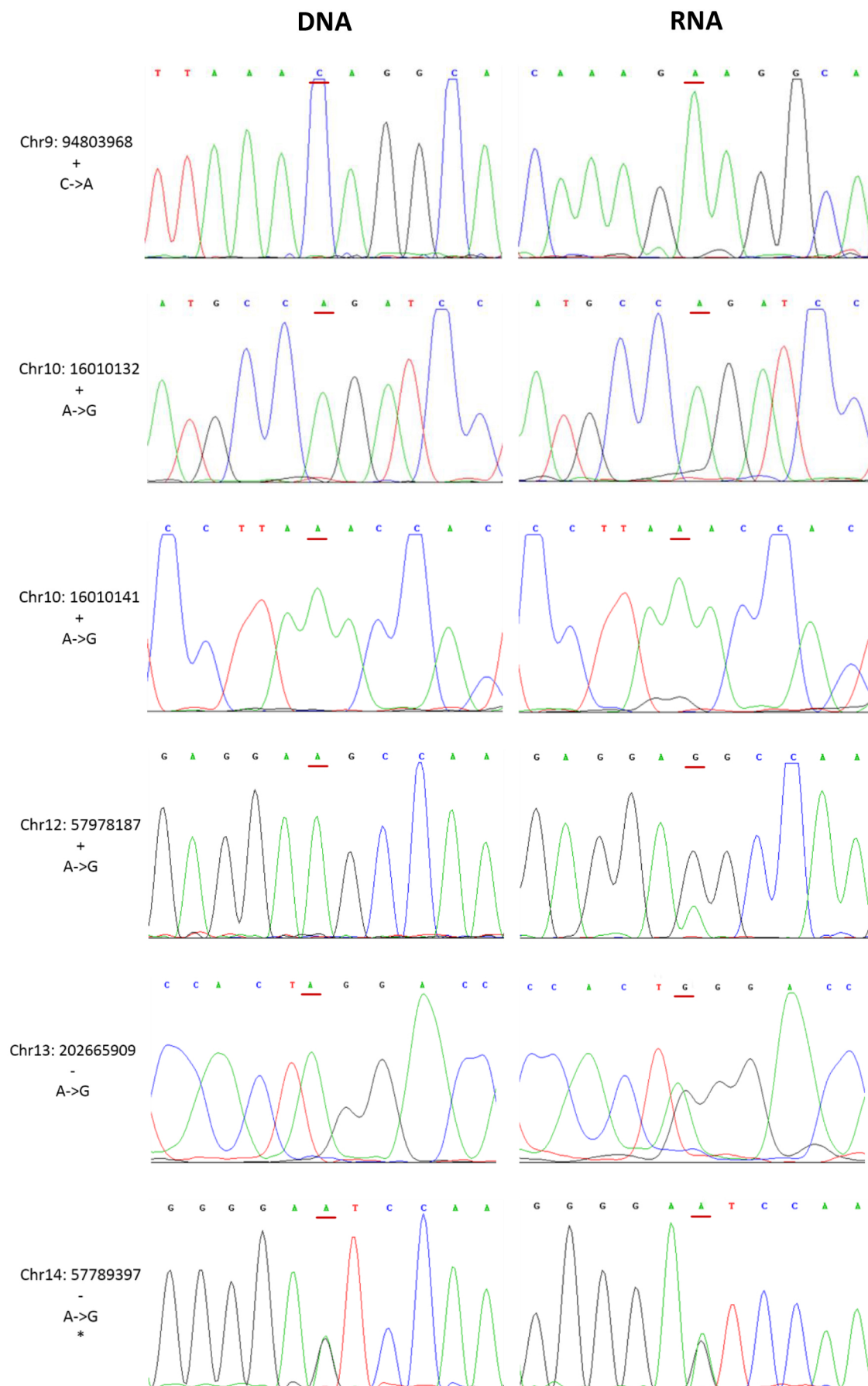

S2-9

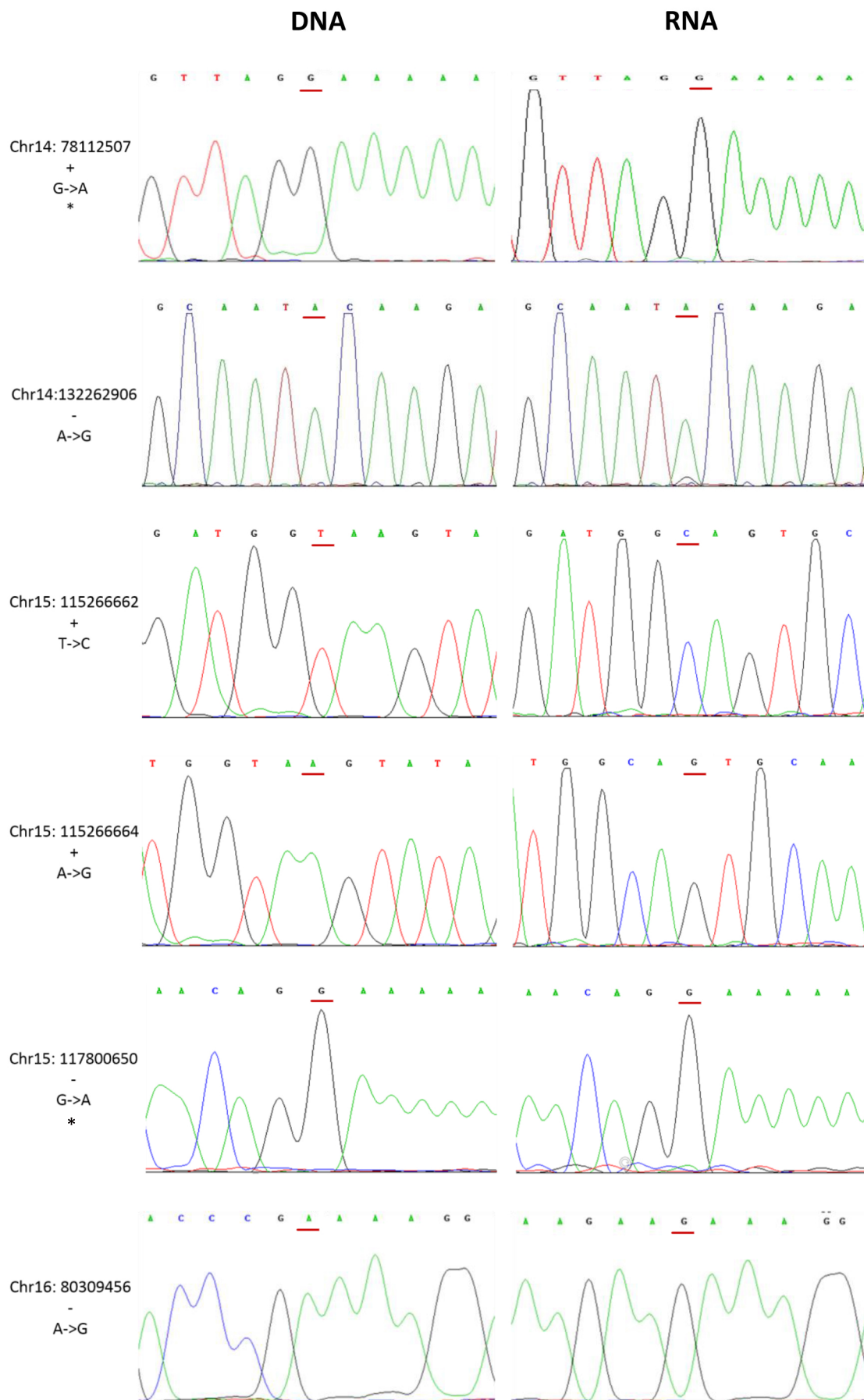

## S2-10

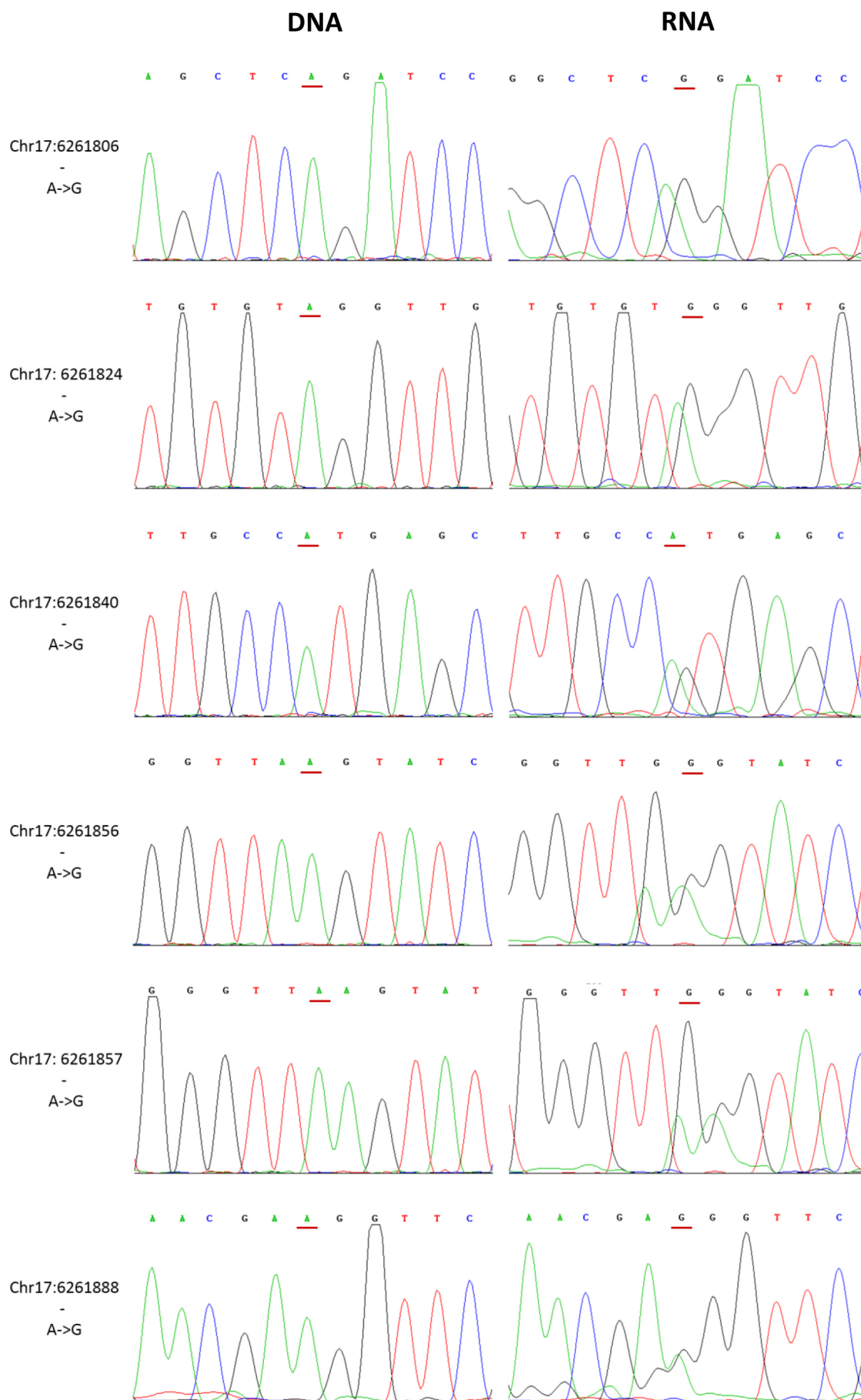

S2-11

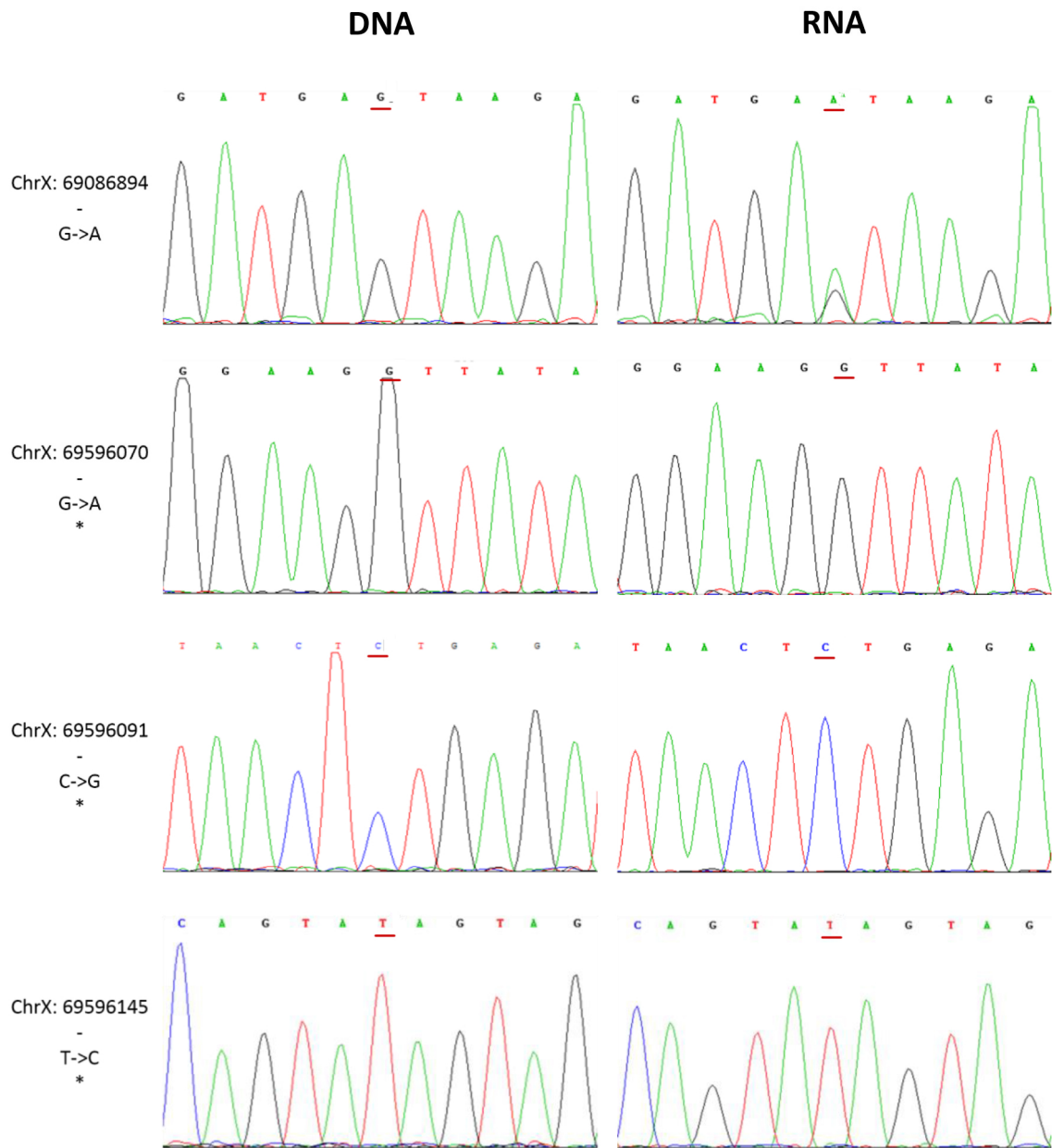

**Figure S3**

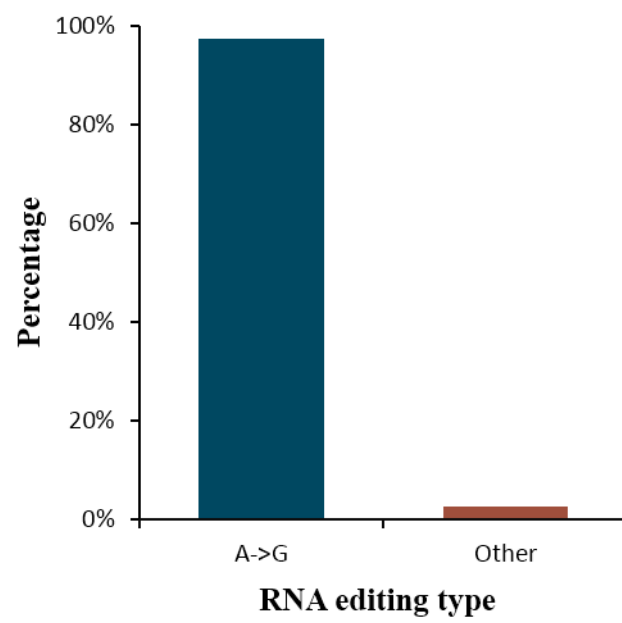

**Figure S4**

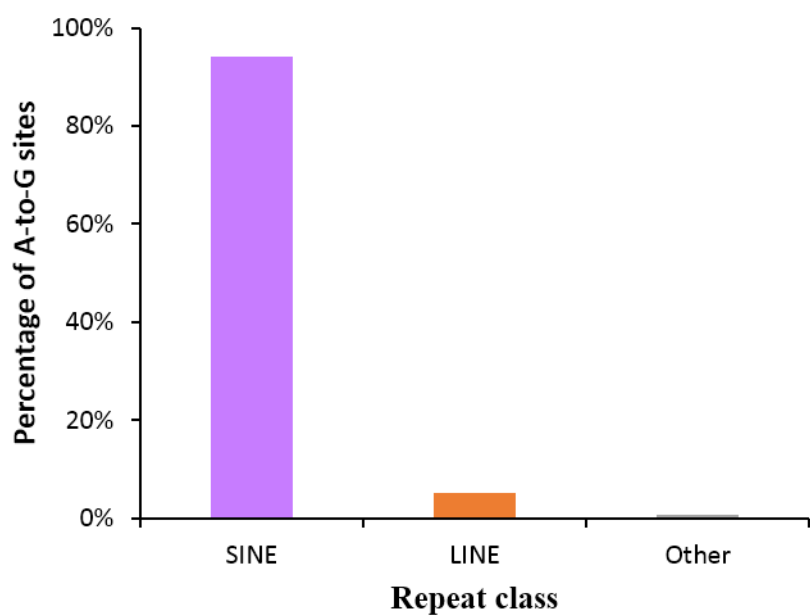

Figure S5

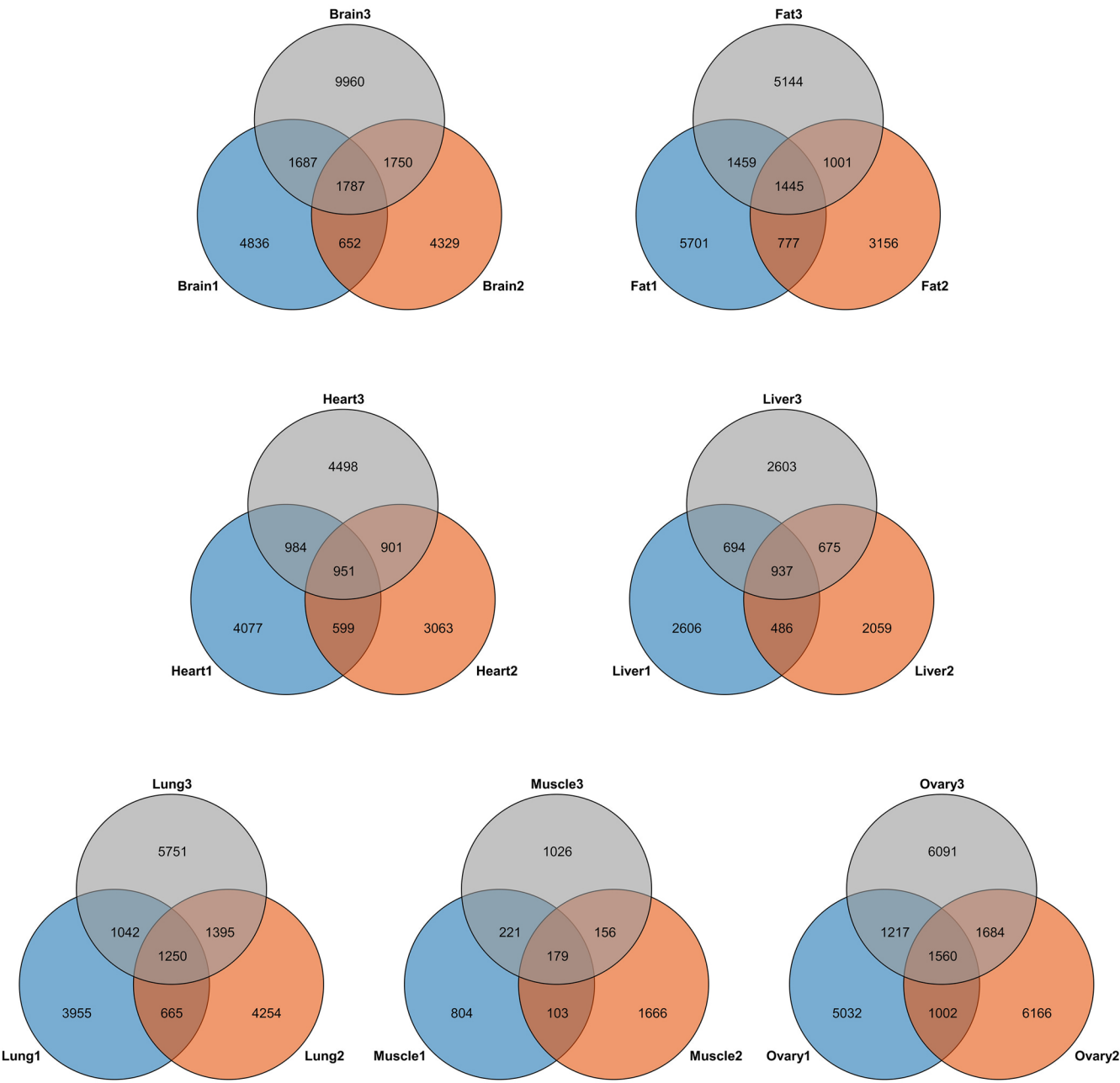

**Figure S6**

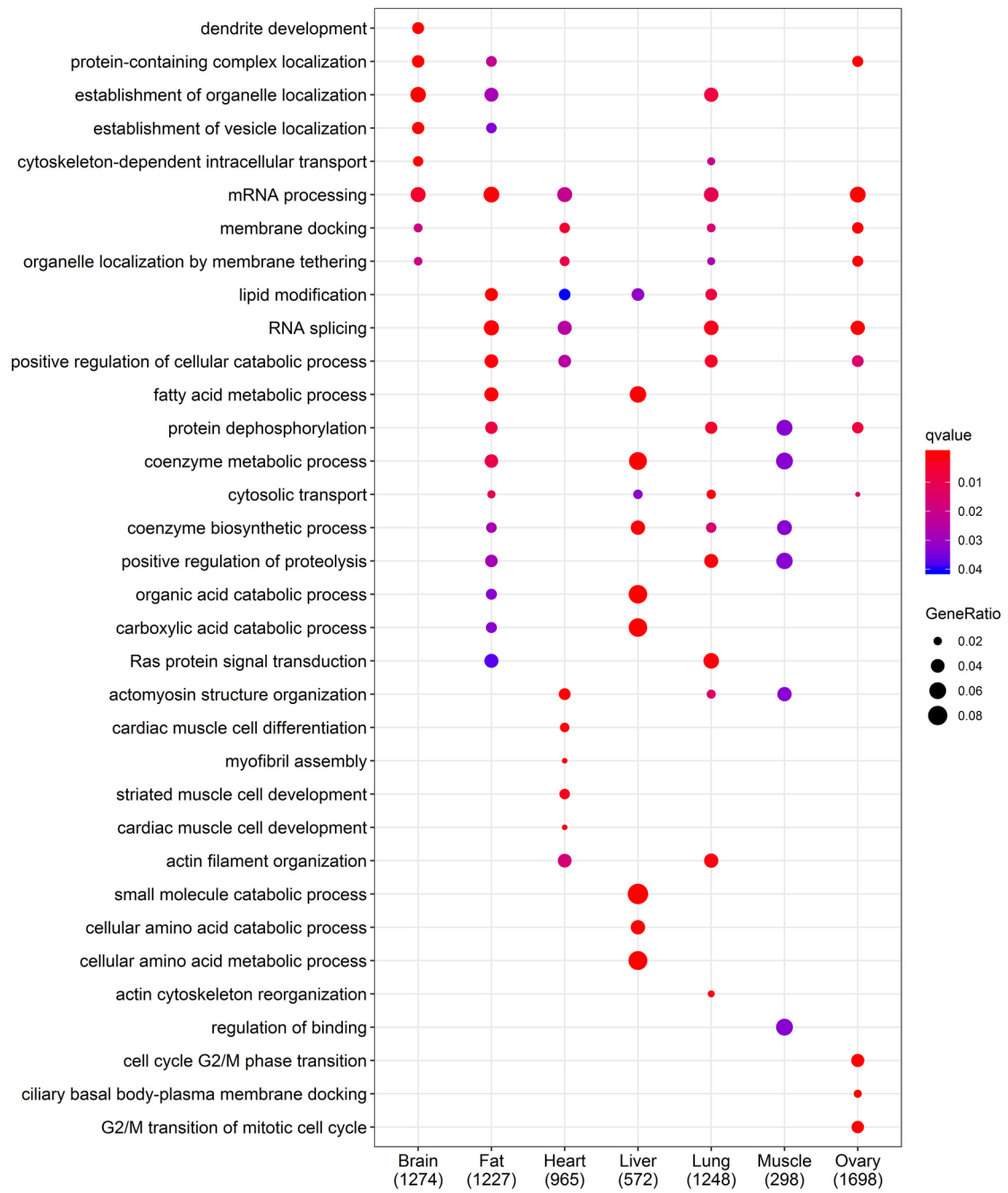

**Figure S7**

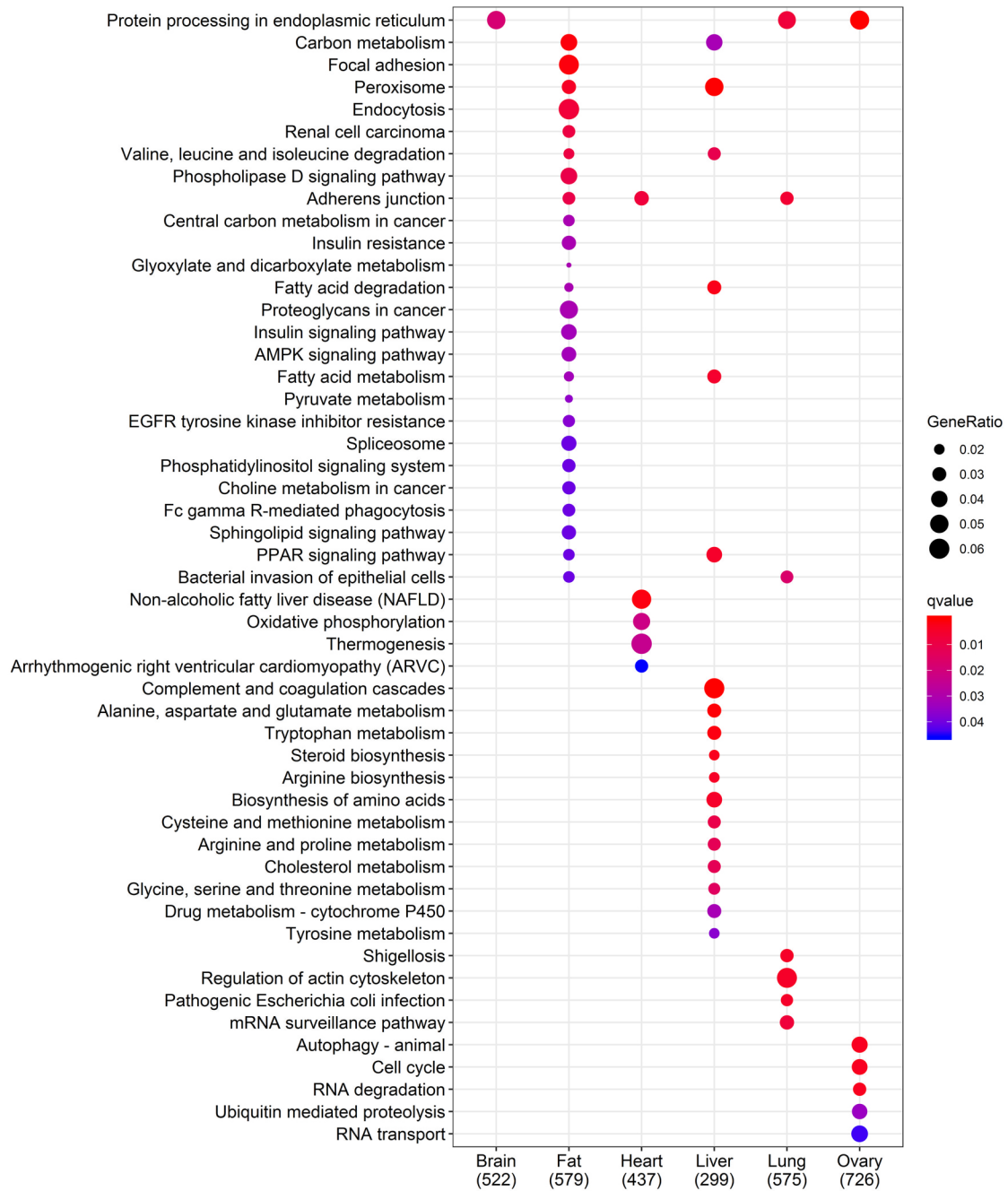

Figure S8

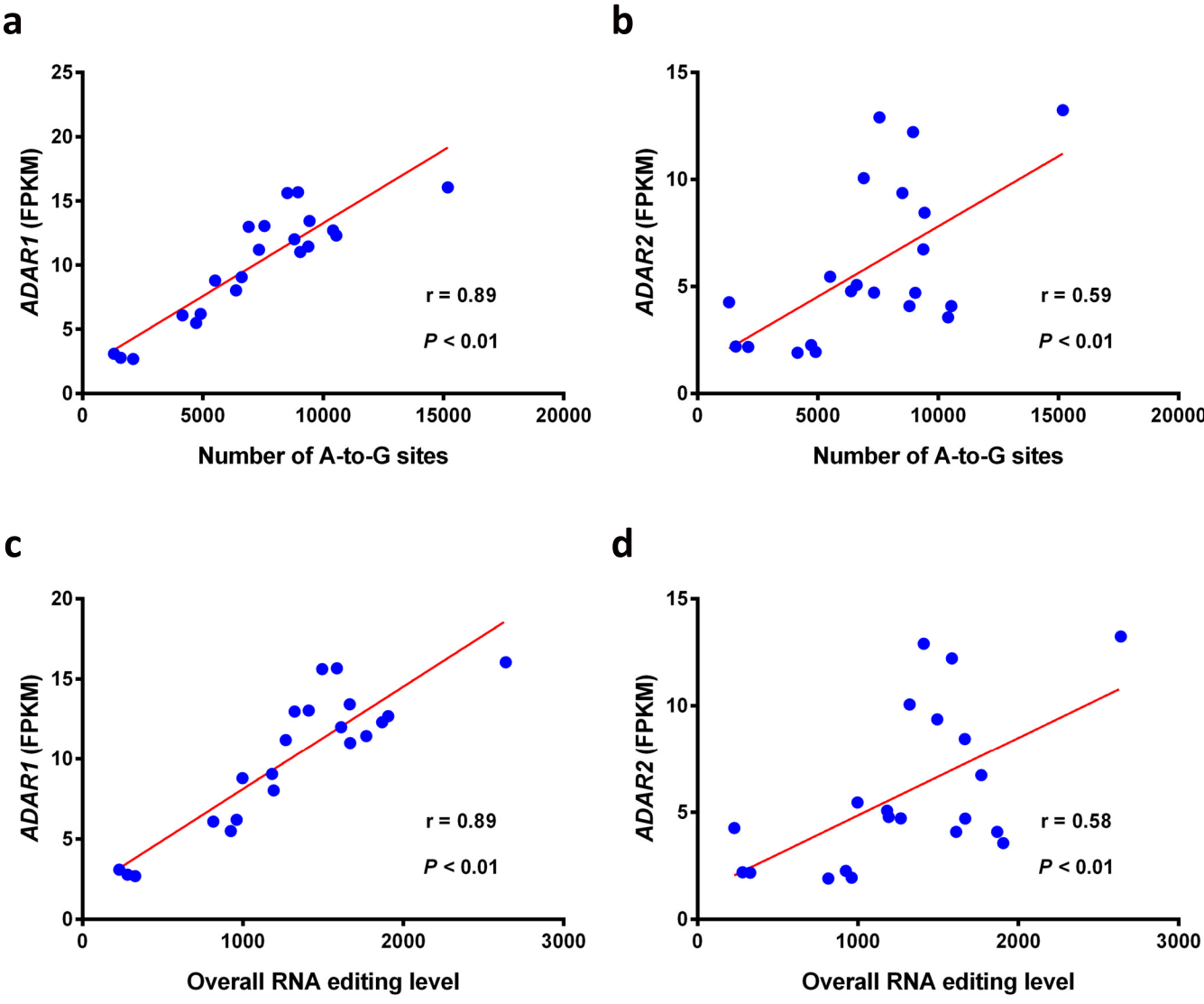

Figure S9

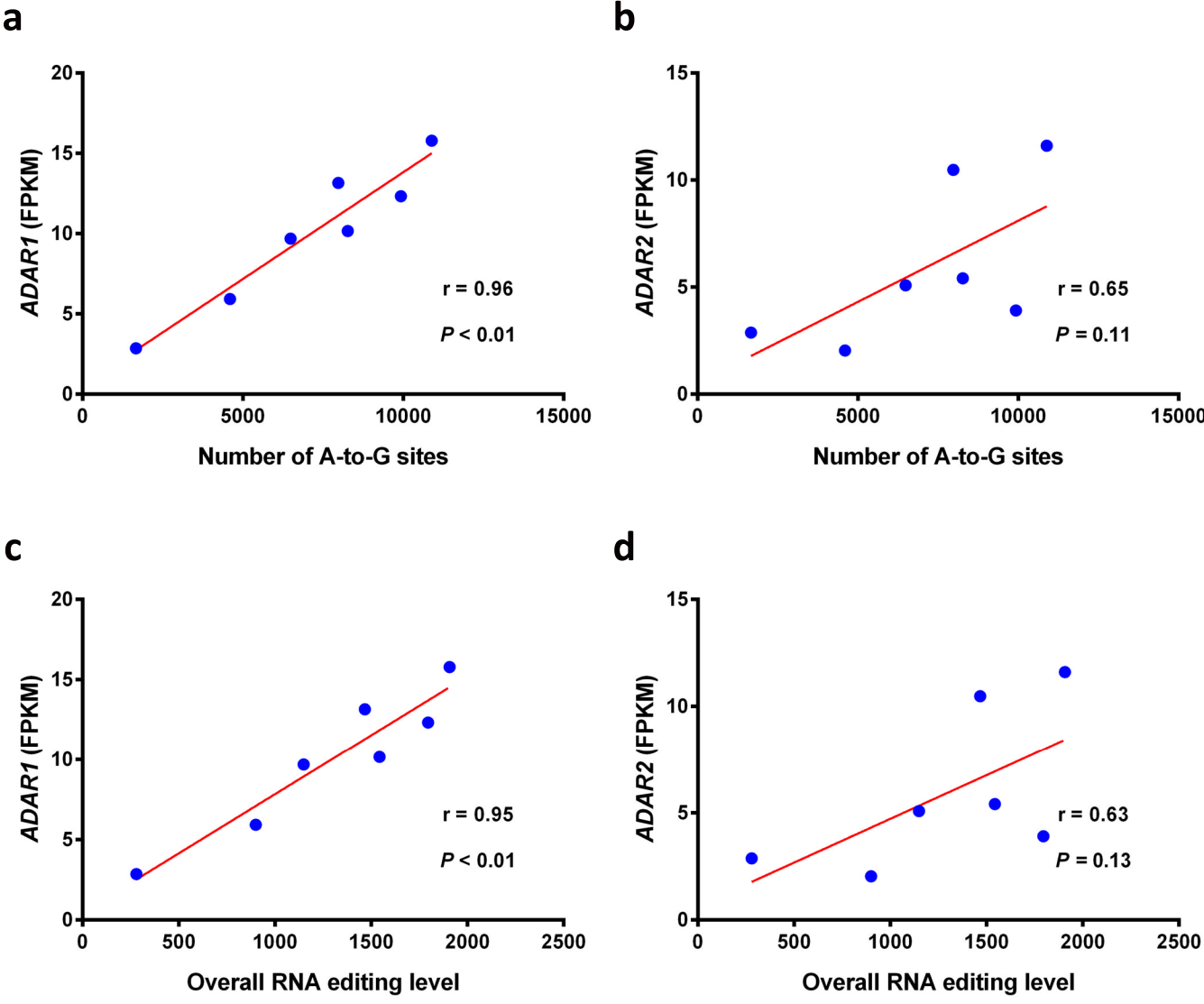

Supplement: Supplementary file 1 — Figure S1. Overview of the experimental design. Figure S2. Results of the Sanger sequencing validation of all 64 selected editing sites. For each candidate editing site (indicated by genome coordinates and red arrow), raw chromatograms of sequences derived from cDNA and matched DNA samples are shown. Unverified sites are marked with an asterisk. Figure S3. Distribution of RNA editing types in repetitive sequences. Figure S4. Distribution of A-to-G sites across major repeat families. Figure S5. Overlap of RNA editing sites among the three samples of each tissue. Figure S6. Dot plot of the top five enriched GO biological process terms in each tissue. Dot color indicates statistical significance of the enrichment (q-value); dot size represents the fraction of genes annotated to each term. Figure S7. Dot plot of the enriched KEGG pathways after removing the tissue-specific expressed genes. Dot color indicates the statistical significance of the enrichment (q-value); dot size represents the fraction of genes annotated to each term. Figure S8. RNA editing vs expression of ADARs in all samples. a) We correlated the number of A-to-G sites and ADAR1 expression levels in all samples (n = 21). b) We correlated the number of A-to-G sites and ADAR2 expression levels in all samples (n = 21). c) and d) We calculated the correlations between the overall RNA editing levels and the expression values of ADAR1 and ADAR2 in all samples (n = 21). Correlation coefficients (r) and P-values are shown in each graph. Figure S9. RNA editing vs the expression of ADARs in tissue groups. a) We correlated the number of A-to-G sites and ADAR1 expression levels in all tissue groups (n = 7). b) We correlated the number of A-to-G sites and ADAR2 expression levels in all tissue groups (n = 7). c) and d) We calculated the correlations between the overall RNA editing levels and the expression values of ADAR1 and ADAR2 in tissue groups (n = 7). Correlation coefficients (r) and P-values are displayed in ea [file 40104_2019_326_MOESM1_ESM.pdf]
